# When the wrong metal stops the cycle: Dynamics and specificity in plant copper-dependent peptide cyclases^☆^

**DOI:** 10.1016/j.jinorgbio.2025.113201

**Published:** 2025-12-19

**Authors:** Courtney M. Petersen, Winston C. Pitts, Shabnam Hematian

**Affiliations:** Department of Chemistry, Virginia Tech, Blacksburg, Virginia 24061, USA

**Keywords:** BpC (BURP-domain peptide cyclase), Copper enzymes, Oxidative catalysis, Metalloprotein expression

## Abstract

Herein, we report the expression of split BURP domain peptide cyclases (BpCs), primarily CamB1 from *Ceanothus americanus*, in *Escherichia coli* using the pET22b vector without a fusion partner while retaining their disordered N-terminal region. To our knowledge, this represents the first full-length split BpC expressed and isolated without reliance on a stabilizing fusion tag (*e.g.*, maltose binding protein, MBP). Both CamB1 and ArbB2, from *Coffea arabica*, were purified and refolded from inclusion bodies, and displayed robust catalytic turnover on a minimal peptide substrate. Copper titrations revealed that catalytic assays using glutathione as the reducing agent require copper far in excess of the stoichiometric number of active sites, with activity plateauing at ~50-fold excess, likely reflecting competition with nonspecific copper binding or solution speciation. Using ascorbic acid in place of glutathione not only restores but also increases maximal activity, requiring only near-stochiometric copper. Metal impact studies demonstrated that noncognate metals inhibit activity. Zn(II) most severely inhibited BpC function at low micromolar concentrations in enzyme-initiated assays containing Cu(II), Zn(II), and glutathione, but this effect was markedly alleviated in reducing agent-initiated assays and instead resembled the modest inhibition by Ag(I), which fully suppressed activity only near 1 mM. These results highlight how assay order influences metal competition at the active site. Given that BURP-domain proteins are implicated in plant stress responses, including tolerance to metal exposure, these findings suggest that Zn(II) and Ag(I) inhibition may represent a biochemical mechanism by which environmental metal stress modulates BpC activity.

Copper is an essential redox-active cofactor in plants, supporting electron transport in photosynthesis and oxidative phosphorylation, as well as driving aerobic metabolic pathways such as lignin, alkaloid, flavonoid, lignan, and cyclic peptide biosynthesis [1–4]. These copper enzymes bind dioxygen (O_2_), effecting partial (2 ē) or complete (4 ē) reduction to catalyze oxidative transformations vital for plant growth, structural integrity, and defense [5,6]. Recently identified BURP domain peptide cyclases (BpCs) catalyze copper-O_2_-mediated radical coupling to form a C-C, C-N, or C-O bond between a redox-active aromatic residue (tyrosine, tryptophan, or histidine) and an unactivated C-H bond on another residue. This cross-linking produces macrocyclic structures in plant proteins and ribosomally synthesized and post-translationally modified peptides (RiPPs) [7–10].

While many bacterial enzymes such as cytochromes P450 (P450s) [11,12] and members of radical *S*-adenosyl-l-methionine (rSAM) superfamily [13–16] rely on iron cofactors to install such cross-links, BpCs and fungal enzymes containing domain of unknown function (DUF) 3328—also recently discovered to be copper-dependent—uniquely use copper-O_2_ chemistry to catalyze the biosynthesis of these cross-linked peptides [10,17–19]. It is worth noting that these emerging classes of copper-dependent enzymes represent a rapidly expanding field. In the DUF3328 family, functions now extend beyond peptide cyclization to include C(*sp*^*3*^)–H bond halogenation [20] and hydroxylation [21]. This diversification closely parallels our proposal that, beyond BpCs, other BURP domain proteins may catalyze additional oxidative reactivities yet to be uncovered [10].

The BURP domain is a ~230-residue C-terminal module containing conserved sequence motifs—an FF dipeptide, a surface CX_11–12_C pair (CC-S), and four CH repeats (CHX_10_CHX_25–27_CHX_25–26_CH)—whose histidines (HH-A and HH-B) colocalize to provide the coordination environment for two copper sites (Fig. 1). Sequence diversity outside the domain underlies distinct subclasses, ranging from short N-terminal linkers to long tandem repeat insertions. Genomically, BpCs occur as fused or autocatalytic systems (*e.g.,* AhyBURP, KjaBURP, SkrBURP), where the substrate peptide is encoded within the same gene, or as split systems with separate genes (*e.g.,* ArbB2, ArbB3, CamB1) [10,22,23].

The first crystal structure of a BpC, from a fused AhyBURP system of the USP subclass, was resolved to 3.2 Å and revealed a half-holo AhyBURP dimer with Cu(II) occupying the C-terminal Cu_B_ site while the N-terminal Cu_A_ site was vacant [10,24]. Both sites can accommodate type 2 (T2) copper centers, which are typically three- or four-coordinate with histidines and a water or hydroxyl ligand, lack intense electronic absorption, and display A_‖_ > 140 × 10^−4^ cm^−1^ [6]. Three conserved cysteine pairs (CC-S, CC-A, and CC-B) may serve as an electron relay, engaging in dithiol/disulfide exchange to deliver electrons (and protons) from exogenous reductants to the active site before O_2_ binding [10]. This structural context is critical, as BpC activity could be highly sensitive to competing metal ions, which can inhibit function by displacing copper, disrupting active-site geometry, or interfering with electron transfer; in some cases, such metals may also subtly enhance or regulate activity [25].

In recombinant expression of BpCs in *E. coli* for *in vitro* studies, all but two—fused AhyBURP and split ArbB3 BpCs—have been expressed with a truncated N-terminus (Table S1), often replaced or fused to solubilizing and stabilizing tags such as MBP (maltose binding protein) [18,23,26,27]. Consequently, the functional role of the unstructured N-terminal region in relation to the BURP domain remains unclear, particularly given its variation in length across split BpCs (*e.g.*, short in CamB1, long in ArbB2, Fig. 1). This structural and expression context is critical for interpreting both *in vitro* activity and the enzyme’s functional role *in vivo*.

In this study, we focus on the split BpC CamB1 from *C. americanus* and use ArbB2 from *C. arabica* as a benchmark to compare expression, activity, and copper loading. MBP-fusion constructs of both enzymes have been characterized previously, with ArbB2 studied in greater detail [26]; thus, we concentrate our metal impact studies on MBP-free CamB1. To this end, we expressed both enzymes in the pET22b vector with preserved N-termini and examined the effects of Zn(II) and Ag(I) on catalytic performance, providing insights into BpC metal specificity and/or inhibition.

pET22b is a ~5.5 kbp plasmid that incorporates the 22-residue N-terminal leader sequence of pectate lyase B (pelB), a hydrophobic signal peptide that directs recombinantly expressed proteins from the cytoplasm to the periplasm. This approach can offer several advantages: first, many enzymes that utilize copper catalytically are found extracellularly or in the periplasm, likely reflecting the tight regulation of free copper ions in the cytoplasm [28]; additionally, previous studies have shown that the proper folding of copper enzymes with internal disulfide bonds, similar to those observed in BpCs (*e.g.*, human peptidylglycine α-hydroxylating monooxygenase, PHM), is critical for function [29,30]. The fewer proteases and oxidizing environment of the periplasm, together with its dedicated enzymatic machinery for disulfide bond formation [31,32], may, therefore, be better suited than the reducing environment of the cytoplasm for achieving the favored redox state of the three conserved cysteine pairs (CC-S, CC-A, and CC-B) found in BpCs (Fig. 1) [10].

Herein, we engineered ArbB2 to retain its full disordered N-terminus (His_6_-ArbB2ΔV21), in contrast to the truncated construct MBP-ArbB2ΔM75 (hereafter referred to as MBP-ArbB2) that was previously characterized [26]. Full-length ArbB2 could be expressed successfully (Fig. S1) using pET22b; however, this did not yield soluble protein to any appreciable extent. Consequently, ArbB2 was isolated, purified, and refolded from insoluble inclusion bodies using a method similar to that employed for AhyBURP and MBP-ArbB2 (though MBP-ArbB2 was refolded from soluble aggregates) [24,26]. Size exclusion chromatography of refolded ArbB2 revealed the presence of multiple oligomeric states (Fig. S2), with the most active fractions corresponding to an elution volume consistent with monomeric protein (predicted 36 kDa; experimental 34.8 kDa, Figs. S2 and S3). While this result aligns with the earlier finding that the active form of MBP-ArbB2 is monomeric, the maximum turnover number achieved by ArbB2 in our *in vitro* end-point assay (23.5 ± 1.3 using the minimal substrate ArbA2_20mer_-FLWGY, Fig. S4) was substantially lower than that observed for MBP-ArbB2 (~110) using the same substrate and assay conditions [26].

Importantly, an analogous protocol using pET22b could be employed to obtain full-length His_6_-CamB1ΔN23, hereafter referred to as CamB1. As with ArbB2, expressed CamB1 necessitated purification and refolding from insoluble inclusion bodies. During the optimization of expression and purification of CamB1, we consistently observed a slightly larger protein (~33 *vs.* 31 kDa, CamB1’, Fig. S5A) that co-purified with CamB1. In-gel digestion followed by mass spectrometry (MS) analysis confirmed this species to be an alternative form of CamB1 (Figs. S6–S8; Tables S4–S5). This form comprised roughly 50 % of the total protein obtained after purification and was determined to be catalytically inactive based on turnover experiments performed with His_6_-tag-free CamB1 (Fig. S5B, Table S2). The ~2 kDa higher mass is consistent with pelB-bound CamB1, in which the highly hydrophobic leader peptide (~2.2 kDa) was not cleaved. Such incomplete processing likely reflects saturation of the Sec translocon under the very high expression levels observed (~10–12 mg of protein per gram of wet cell pellet), preventing efficient cotranslational cleavage of the signal sequence. By contrast, refolded full-length CamB1 was catalytically active, achieving 29.9 ± 2.60 turnovers with the minimal substrate CamA1_20mer_-ILLY (Fig. S9), compared to the 85 turnovers previously reported for MBP-CamB1 [26]. The lower turnover numbers observed for both ArbB2 and CamB1 could arise from a regulatory role of the N-terminus or from subtle differences in aggregation behavior of split BpCs without MBP. AlphaFold models suggest that the N-terminus can occlude the active site but shifts away upon substrate binding, hinting at a role in gating substrate access (Fig. S10). Further studies are needed to define how N-terminal dynamics influence catalytic efficiency in native *vs.* MBP-fused BpCs.

In previous *in vitro* assays, BpCs were tested under conditions of excess copper and reducing agents [18,23,26,27]. In particular, MBP-ArbB2 was systematically screened with different reductants and showed a strong preference for glutathione (GSH), and those optimized conditions were then used for MBP-CamB1 [26].

Building on these observations, we next examined how copper concentration influences enzyme activity. The precise copper loading required for catalytic turnover has not yet been established for any split systems; however, prior work on autocatalytic AhyBURP demonstrated that a 2-fold molar excess of copper relative to available binding sites was necessary for substantial conversion of linear peptide to bicyclic product, while a ~50-fold excess was required for two complete turnovers, *i.e.*, full consumption of cores [24]. Variants lacking any of the four key histidine residues are completely inactive in both autocatalytic and split systems, highlighting the essential role of both copper centers in catalysis [24,26].

In our copper titrations, ArbB2 reached maximal turnover only at ~100-fold copper excess, whereas CamB1 required substantially less (~40-fold excess) (Fig. 2). Therefore, while not a direct measure of binding affinity, our results suggest that the apparent Cu(II) dissociation constant(s) may vary significantly across different BpCs. Notably, T2 copper centers are generally characterized by relatively weak to moderate affinity (*K*_d_ range from 10^−5^ to 10^−9^ M) and are often partially depleted during *in vitro* assays [33–37]. Under our conditions, with excess reductant (500 μM GSH), rapid Cu(II) reduction and formation of Cu(I)–GSH complexes could act as molecular chaperones, mediating weaker Cu(I) binding to the enzyme and thereby making metal incorporation into the apo-enzyme less thermodynamically favorable [38,39]. Here, we often use the general term *copper loading* for convenience but mechanistically interpret our data in a framework where Cu(II) binds first and is reduced to the catalytically active Cu(I) state under assay conditions.

Next, we assessed the effects of Zn(II) and Ag(I) ions on CamB1 activity. Metal competition and substitution experiments have long been used to probe copper’s catalytic role in metalloproteins. BURP domain proteins are also implicated in diverse aspects of plant growth and stress tolerance, including heavy metal stress, although their molecular mechanisms remain poorly understood [40,41]. Zn(II), a non-redox-active mimic of Cu(II), can occupy Type 2 or Type 3 copper-binding sites but cannot support oxidative catalysis, thereby blocking turnover [42,43]. By contrast, Ag(I), a soft metal, can mimic Cu(I) and serves as a useful probe for this oxidation state but cannot support catalysis due to its inability to cycle between redox states [33,44,45]. Although higher oxidation states of silver exist (*i.e.*, Ag(II) and Ag(III)), the instability of Ag(II) precludes one-electron transfer chemistry analogous to Cu(I)/Cu(II) in BpCs.

Experimentally, we observed that as little as 60 μM Ag(I) was sufficient to begin inhibiting substrate turnover even under excess copper (150 μM), and a six-fold molar excess of Ag(I) over CuSO_4_ completely abolished activity (Fig. 3A). For MBP-ArbB2, catalysis initiated with Cu(I) consistently resulted in higher substrate conversion than afforded initiating with the Cu(II) state [26]. While Ag(I) inhibition is consistent with the importance of Cu(I) for maximal activity, the thiophilic nature of Ag(I) suggests binding to conserved cysteine pairs, which could block the proposed electron transfer pathways or destabilize conformations critical for catalysis [45,46].

Strikingly, Zn(II) completely inhibits CamB1 turnover at 60 μM, representing over an order of magnitude stronger inhibition than Ag(I) (Fig. 3B). Although zinc could in principle compete with Cu(II) for HH-A and HH-B sites, the Irving-Williams series—which places Cu(II) at the peak of stability among first-row divalent transition metals—together with the excess copper present (150 μM) makes simple displacement unlikely [47]; however, recent protein-design studies have shown this stability trend can be overridden [48]. This suggests the molecular basis for zinc inhibition could be more complex, potentially involving competition with Cu(I) under reducing conditions, formation of mixed Cu(I)/Zn(II)-GSH species that complicate *in vitro* assays, or secondary binding that interferes with BpC/substrate interactions and catalysis. Supporting this idea, AlphaFold 3 predictions in the presence of both copper and zinc (Fig. S11) indicate a stronger preference for Cu(II) at the Cu_B_ and Cu_A_ sites, respectively, whereas excess zinc appears to associate with surface histidines of both the BpC and its substrate partner.

This raises the possibility that BpCs, like non-type 3 binuclear enzymes such as PHM, dopamine β-monooxygenases (DβM), and tyramine β-monooxygenase (TβM), may employ asymmetrical copper roles, with one site specialized for O_2_ activation (Cu_M_) and the other dedicated to electron transfer (Cu_H_) [49]. One could envision a T2-like center, analogous to Cu_H_ or even a type-0 site—an artificial construct first pioneered by Gray and coworkers [50]—serving to support rapid electron transfer.

To test whether Zn(II) inhibition arises primarily from solution-phase Cu(I)/Zn(II)-GSH speciation prior to metal loading, as suggested by our Cu(II) titrations, we changed the order of addition so that apo-CamB1 was first preincubated with Cu(II) and Zn(II) before initiating the reaction with 500 μM GSH (Fig. 3B). Under these preloading conditions, where Cu(II) can bind the enzyme before reduction, Zn(II) inhibition is markedly attenuated and follows expectations from the Irving-Williams series, with Cu(II) preferentially occupying the *bis*-histidine sites.

Having confirmed the role of Cu(I)/Zn(II)-GSH speciation in Zn(II) inhibition, we next reevaluated the copper loading of CamB1 using an analogous assay initiated by GSH. Interestingly, adding GSH after preincubation of apo-CamB1 with Cu(II) did not restore the copper loading to any significant extent (Fig. 4). A ~40-fold excess of Cu(II) was still required to reach the maximal turnovers, although modest gains in activity were observed at CamB1:CuSO_4_ ratios <50. This slight improvement at lower copper concentrations is consistent with competition between CamB1 and GSH for Cu(II) in the *GSH-initiated* experiments and for Cu(I) in the *protein-initiated* experiments.

In support of the dominant role of the Cu(I)-GSH pool, we replaced GSH with ascorbic acid (AscH) as the reductant, which shifted copper loading to essentially stoichiometric (~2 copper equivalents per monomer) and increased maximal conversion to ~70 turnovers before slightly declining and plateauing near ~60 turnovers at higher copper concentrations (Fig. 4). Recent solution studies on copper/GSH/AscH systems in 50 mM Tris pH 7.5 demonstrated that GSH rapidly reduces Cu(II) to Cu(I) and sequesters it in Cu-GSH clusters, whereas AscH alone maintains an electrochemically competent Cu(II)/Cu(I) couple [51]. Moreover, GSH/GSSG bind copper more strongly than AscH. Together, these observations support a model in which strong copper chelation by GSH draws copper into off-enzyme pools, necessitating large Cu excess in GSH-based assays.

Our findings on metal inhibition, recovery, and the preserved N-termini provide complementary insights into BpC function. Prior work established Cu(I) as the catalytically active species, with Cu(II) becoming competent in the presence of reducing agents [24,26]. Here, we show that Ag(I) and Zn(II) disrupt activity through distinct mechanisms, while structural analyses highlight the importance of flexible, diverse N-terminal regions, particularly in split BpCs, for controlling turnover. We further demonstrate that assay order and reductant choice strongly influences metal competition at the active site, with formation of Cu(I)-GSH complexes increasing the overall copper requirement for catalysis and limiting total turnovers. In line with these observations, substitution of GSH with AscH not only increased the number of turnovers observed but also decreased the copper loading needed for maximal activity to near the expected stoichiometric values. Together, these results suggest that BpC reactivity depends on both metal-site integrity and dynamic N-terminal elements to regulate catalysis.

Future studies of electron transfer pathways, metal binding, and substrate interactions across redox states will clarify how BpCs differ from other copper-based oxygen-activating and reducing enzymes. More broadly, this work provides a framework for probing how heavy metals such as silver (*e.g.*, silver nanoparticles) and zinc induce oxidative stress in plants and how environmental metal availability shapes both symbiotic and phytotoxic outcomes.

## Supplementary Material

MMC2

MMC1

## Figures and Tables

**Fig. 1.**
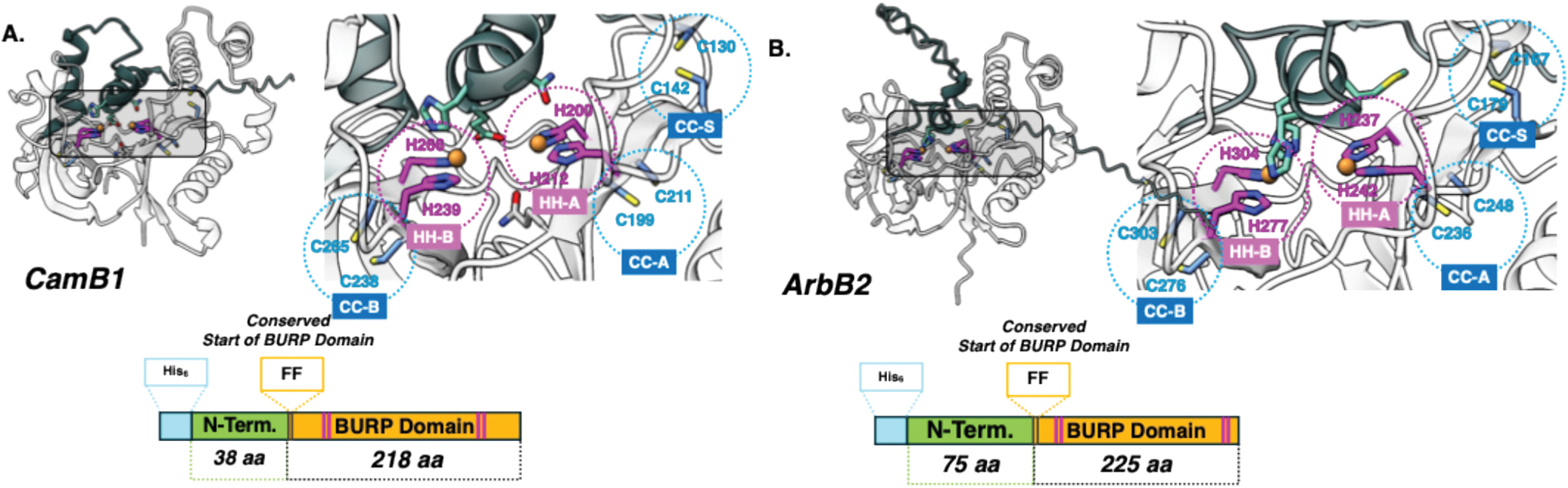
AlphaFold-predicted structures of (**A**) holo-CamB1 and (**B**) holo-ArbB2, with insets highlighting the two CuHis_2_ binding sites designated as Cu_A_ (N-terminal site) and Cu_B_ (C-terminal site) and their respective cysteine pair. For clarity, the respective N-termini of full-length ArbB2 and CamB1 are shown in green. Schematic construct diagrams shown below each structure illustrate the domain organization and boundaries of key regions within the protein sequences. Copper is shown as bronze spheres.

**Fig. 2.**
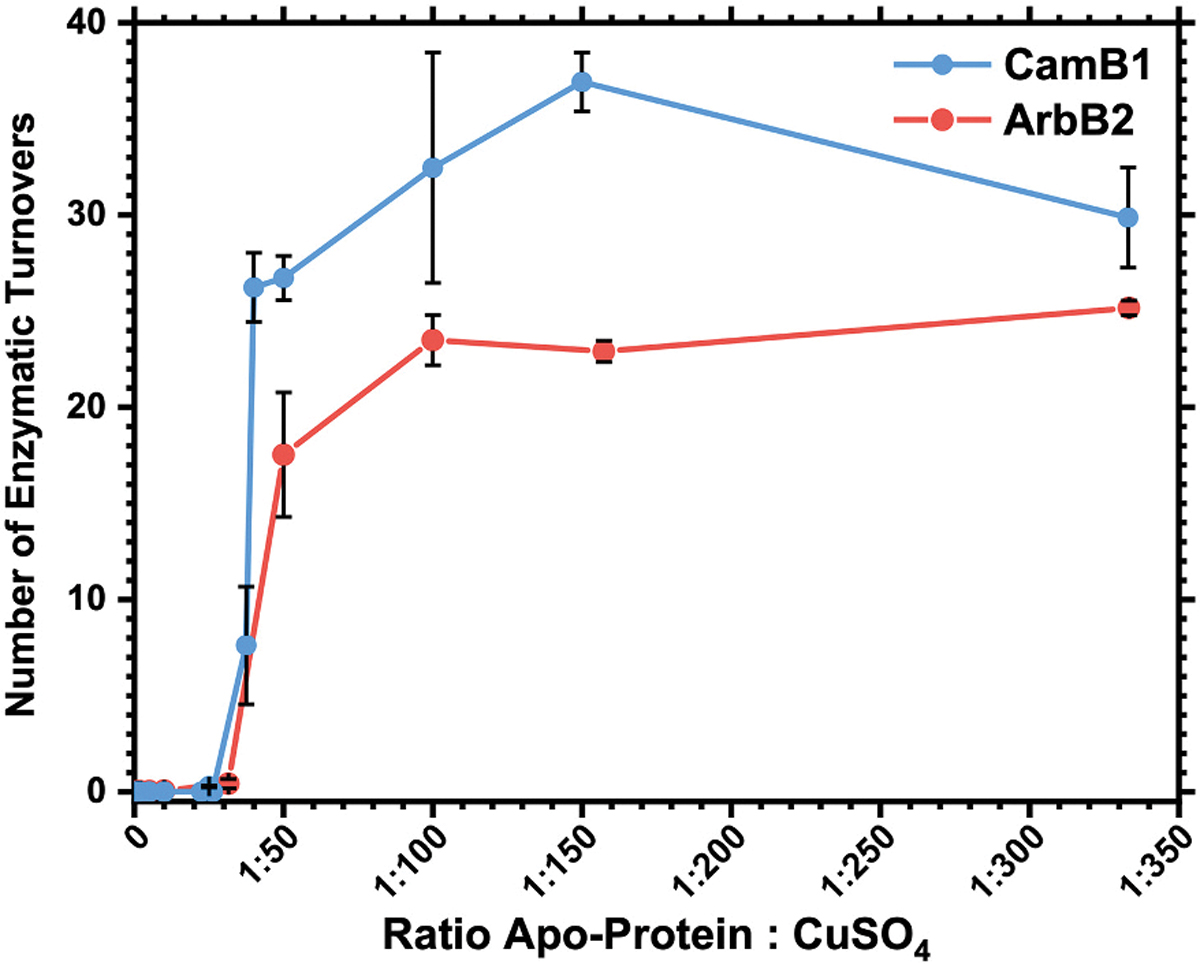
Copper titrations of BpCs in the presence of 500 μM GSH and the corresponding substrate peptide (150 μM) in 50 mM citrate/phosphate, pH 8.0. Substrate cyclization was quantified by LC-MS (see SI). Each data point is the average of triplicates, and error bars represent one standard deviation.

**Fig. 3.**
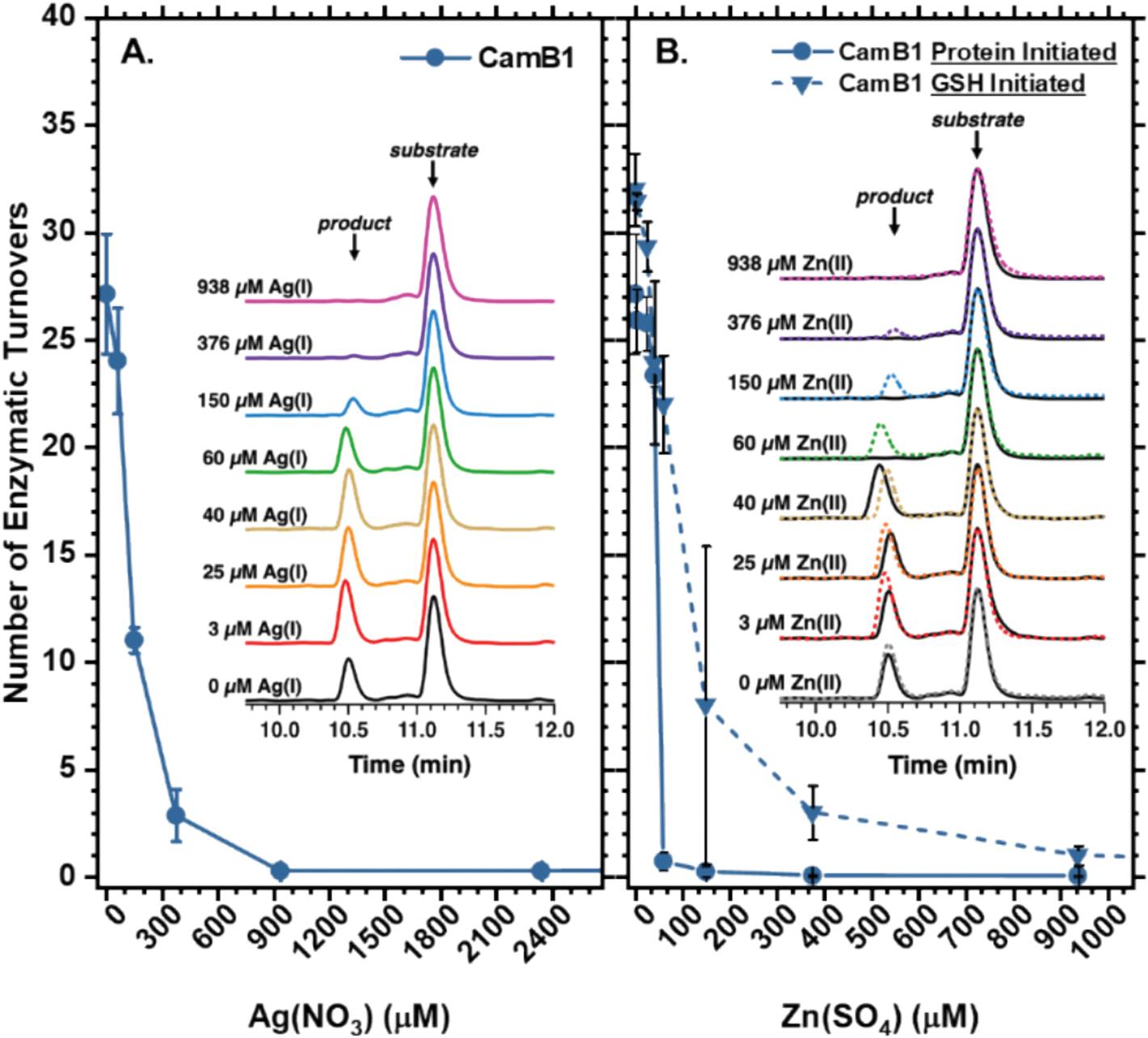
LC-MS analysis of CamB1 inhibition by (**A**) Ag(I) and (**B**) Zn(II). Titrations in 1.5 μM active CamB1, 150 μM CuSO_4_ in 50 mM citrate/phosphate pH 8.0. Solid and dotted lines indicate data from *protein-initiated* and *GSH-initiated* assays, respectively. Each data point is the average of triplicates and error bars represent one standard deviation.

**Fig. 4.**
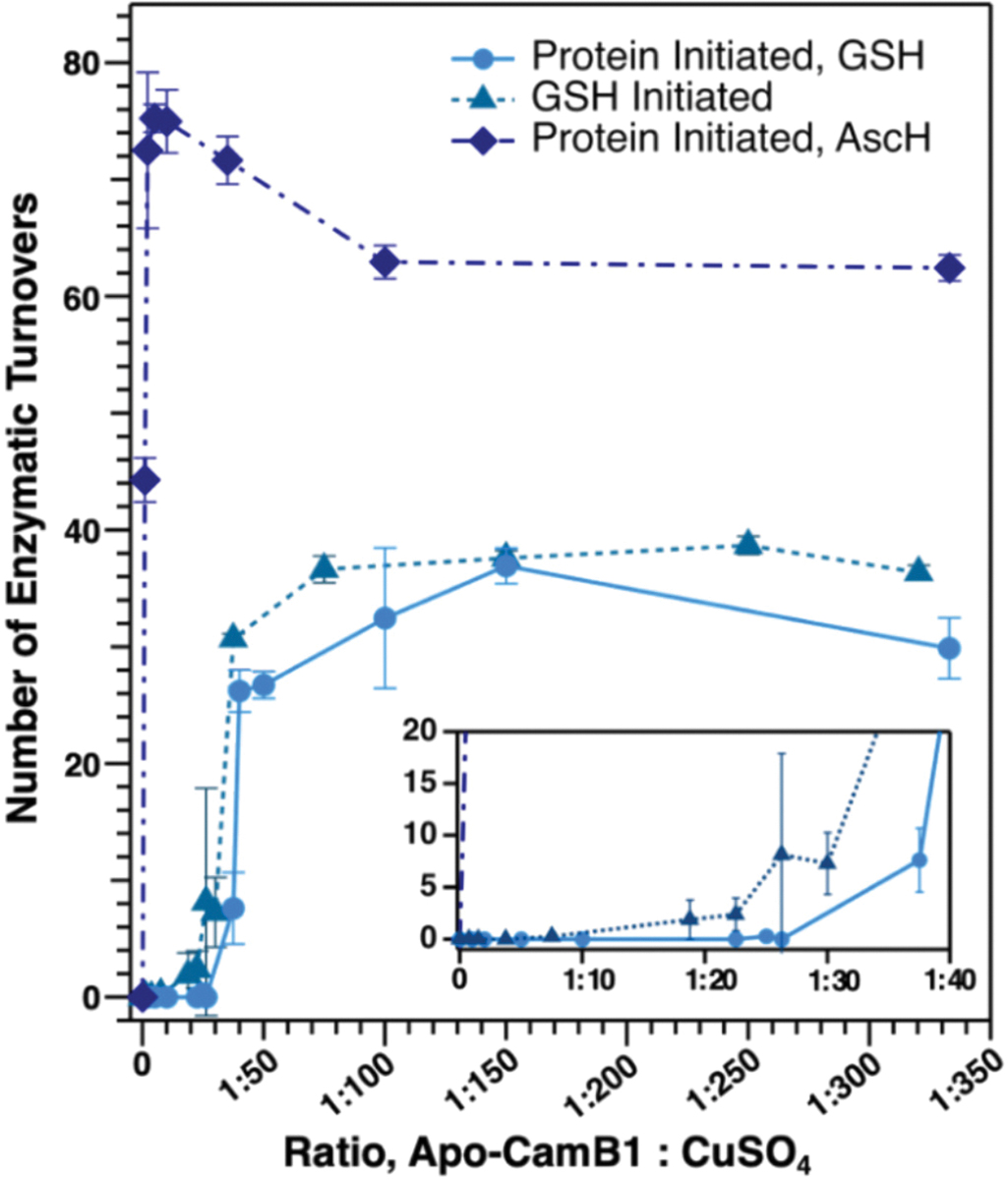
The influence of reductant choice and initiation reagent on the copper loading of CamB1. *Inset:* number of CamB1 turnovers observed below a forty-fold excess of CuSO_4_. All reactions were completed with 500 μM reductant (GSH or AscH) and 150 μM CamA1_20mer_-ILLY in 50 mM citrate/phosphate, pH 8.0. Substrate cyclization was quantified by LC-MS, where each data point is the average of three replicates. Error bars represent one standard deviation.

## Appendix A. Supplementary data

Supplementary data to this article can be found online at https://doi.org/10.1016/j.jinorgbio.2025.113201.
