# Supplementary material for: When the wrong metal stops the cycle: Dynamics and specificity in plant copper-dependent peptide cyclases
Source: J Inorg Biochem. Author manuscript; Available in PMC 2026 Feb 6. (PMC12874468; doi:10.1016/j.jinorgbio.2025.113201)
Supplement: MMC2 [file NIHMS2135269-supplement-MMC2.docx]

***Supporting Information***

*When the wrong metal stops the cycle: Dynamics and specificity in plant copper-dependent peptide cyclases*

Courtney M. Petersen, Winston C. Pitts, and Shabnam Hematian*

Department of Chemistry, Virginia Tech, Blacksburg, Virginia 24061, United States

**Table of Contents:**

1. General Materials............................................................................................................**S2**
2. General AlphaFold 3 Modeling........................................................................................**S2**
3. Protein Expression .........................................................................................................**S2**
4. Protein Purification..........................................................................................................**S3**
5. Gel Filtration Calibration…..............................................................................................**S5**
6. Metal Titrations…............................................................................................................**S5**
7. UHPLC-HRMS Method .............…..................................................................................**S6**
8. Peptide Mass Fingerprinting (PMF) by In-gel Digestion and LC-MS/MS……………………**S6**
9. DNA, Protein, and Peptide Sequences with Accession Numbers…………………………….**S8**
10. Supplemental Figures……………………………………………………………………......**S11**
11. Supplemental Tables…………………………………………………………………….…...**S22**
12. References……………………………………………………………..........………………..**S27**

**Materials and Methods**

**General Materials**

Terrific broth (#T15100) and CAPS (3-(cyclohexylamino)propane-1-sulfonic acid, #C30060) were purchased from Research Products International. Betaine hydrochloride (#29816), arginine (#00024), and arginine hydrochloride (arginine-HCl, #00331) were purchased from Chem Impex International. Imidazole (#37864) was purchased from Honeywell. l-ascorbic acid (#795437), silver nitrate (#209139), zinc sulfate monohydrate (#307491), potassium phosphate monobasic (#PX1562-5), and Supelco® 0.1% (v/v) formic acid in acetonitrile were purchased from Sigma-Aldrich. Tris Base (tris(hydroxymethyl)aminomethane, #BP152-1), potassium phosphate dibasic (#BP363), glycerol (#BP229), ampicillin sodium salt (#BP1760), LB broth (#BP1426), and formic acid (#A117) were purchased from Fisher Scientific. l-glutathione, reduced (#J62166.14), N-laurylsarcosine sodium salt (#434371000), urea (#036428-A3), and Pierce EDTA-free protease inhibitor tablets (#A32965) were purchased from ThermoFisher Scientific. Citric acid (#0529), cupric sulfate pentahydrate (#0330), sodium chloride (#BDH9286), and *β*-mercaptoethanol (#M131) were purchased from VWR. PROTEINDEX™ HiBond™ NiNTA Agarose 6FF resin (#11-0225-100) was purchased from Marvelgent Biosciences, and the peptide substrates ArbA2_20mer_-FLWGY and CamA1_20mer_-ILLY were purchased from Biomatik (Kitchener, Ontario, Canada).

**General AlphaFold 3 Modeling**

AlphaFold 3^1^ was accessed through the website interface. AlphaFold 3 models were generated via multi-seed diffusion sampling without external templates unless noted. For each model, we retained coordinates, per-residue pLDDT, ipTM/pTM (ranking confidence), and PAE. Interfaces were considered reliable only when high ipTM co-occurred with low interface PAE, high interface pLDDT, and support from clustered solutions; otherwise, docking was deemed ambiguous and additional seeds and/or priors were explored. His_6_-ArbB2ΔV21 or His_6_-CamB1ΔN23 sequences were entered as monomers. For substrate-bound models of His_6_-ArbB2ΔV21 or His_6_-CamB1ΔN23, the linear peptide sequences extended-ArbA2_20mer_-FLWGY or extended-CamA1_20mer_-ILLY were included as secondary protein inputs. The exact sequences of extended-ArbA2_20mer_-FLWGY and extended-CamA1_20mer_-ILLY are listed on **pages S8-S10**. Where required, metal ions (Cu^2+^ and/or Zn^2+^) were included as additional inputs. Multiple seeds were evaluated (indexed starting at 1) to assess model robustness.

**Protein Expression**

The ArbB2 and CamB1 constructs used in this paper were designed by first analyzing the native sequences for signal motifs using SignalP 5.0^2^ or SignalP 6.0^3^ as previously described;^4–6^ the signal peptide was removed prior to cloning. Genes encoding residues V21 - I320 of ArbB2 and N23 - N278 of CamB1 (both preceded by protease-cleavable 6×His-tags, see **pages S8-S10**) were codon-optimized for expression in *E. coli* and cloned into pET22b (Millipore Sigma #70765-3, Twist Bioscience, San Francisco, CA) using the NcoI and XhoI restriction sites. The final plasmid constructs were transformed into DH10*β* chemically competent *Escherichia coli* cells for cloning, extracted, and purified using the Monarch® plasmid spin miniprep kit (New England Biolabs) according to the manufacturer’s protocol. Sequence-verified plasmid DNA (Plasmidsaurus) was subsequently transformed into BL21(DE3) chemically competent *E. coli* for protein expression. Prior to expression, 15 mL Lysogeny Broth (LB) cultures containing 100 μg/mL ampicillin were inoculated with single colonies and incubated overnight at 37°C with 220 rpm shaking. The starter culture was then used to inoculate 1 L of Terrific Broth (TB; supplemented with 100 μg/mL ampicillin and 0.4% (*v*/*v*) glycerol) in a 2 L flask. Cells were grown at 37°C, 200 rpm until the OD_600_ measured between 0.5-0.8 at which point cultures were cooled to 18°C for 30 minutes. Protein expression was induced with 1 mM Isopropyl *β*-D-1-thiogalactopyranoside (IPTG) and incubated overnight (18 h for ArbB2, 20 h for CamB1) at 18°C with shaking at 200 rpm. Cells were harvested via centrifugation at 10,000 ×*g* for 10 min at 4°C, and the resulting pellet was stored at −20°C until purification.

**Protein Purification**

ArbB2

The pellet was resuspended in lysis buffer (50 mM Tris Base pH 8.0, 500 mM NaCl, 0.5%(w/v) N-lauroylsarcosine, 10% glycerol, 10 mM *β*-mercaptoethanol (BME), protease inhibitor tablets, EDTA-free) at a 1:6 ratio of wet cell-pellet weight (g):lysis buffer (mL) and lysed using a Branson 150 Sonifier Cell Disruptor, 65% amplitude, 3s pulse on/6s pulse off for a total of 6 minutes (repeated twice). Lysed cells were then centrifuged at 30,000 ×*g* for 15 minutes or until lysate appeared clear. The lysate containing soluble fraction was set to the side, whereas the insoluble pellet was washed with equal volumes of wash buffer (20 mM Tris Base pH 8.0, 500 mM NaCl, 0.25 %(w/v) N-lauroylsarcosine, 500 mM Betaine, 10% glycerol, 10 mM BME) by disturbing the cell pellet until it was resuspended. Following centrifugation at 30,000 ×*g* for 15 minutes, the resulting insoluble pellet was resuspended in equal volumes of solubilizing buffer (8 M Urea, 25 mM Tris Base, 10 mM BME, pH 8.0) and allowed to incubate for 30 minutes. Any remaining insoluble debris was cleared by centrifugation at 15,000 ×*g* for 30 minutes.

The denatured protein was loaded onto a gravity Ni-NTA column (30mL resin bed) that had been equilibrated with Buffer A (8 M urea, 100 mM Tris Base, 100 mM potassium phosphate monobasic, 10 mM BME pH 6.3). Bound protein was then washed with five column volumes (CVs) of Buffer A and eluted with five CVs (15 mL collections) of Buffer B (8 M urea, 100 mM Tris Base, 100 mM potassium phosphate monobasic, 10 mM BME pH 4.5). The pH of elution fractions containing ArbB2 was adjusted to 8.0-8.1, and the eluate was purified a second time on a separate Ni-NTA IMAC column (20mL resin volume) pre-equilibrated with Buffer C (8 M urea, 50 mM Tris Base, pH 8.0, 500 mM NaCl, 10% glycerol, 10 mM BME). The bound fraction was washed with 2×10 mL Buffer C (with 0 mM imidazole) and then 5×10 mL Buffer C (with 30 mM imidazole). The washed protein was eluted using a gradient of 1×10mL Buffer C (with 50 mM imidazole), 3×10 mL Buffer C (with 100 mM Imidazole), 4×10mL Buffer C (with 150 mM imidazole), and 3×10 mL Buffer C (with 250 mM Imidazole). The cleanest 10 mL fractions, as determined by SDS-PAGE, were combined and diluted to a total protein concentration of 0.2 mg/mL with Buffer C (0 mM or 250 mM Imidazole). Refolding of ArbB2 was completed using dialysis under reducing conditions in the following order: Buffer 1 (4 M urea, 50 mM Tris Base pH 8.0, 250 mM NaCl, 10 mM BME, 10% glycerol, and 700 mM arginine-HCl) overnight, Buffer 2 (1 M urea, 50 mM Tris Base pH 8.0, 250 mM NaCl, 10 mM BME, 10% glycerol, and 700 mM arginine-HCl) for 4 hours, Buffer 3 (50 mM Tris Base pH 8.0, 250 mM NaCl, 10 mM BME, 10% glycerol, and 700 mM arginine-HCl) overnight. The refolded protein was then concentrated to ~5 mL using a Vivaspin® 20, 10 kDa MWCO Polyethersulfone (PES) spin concentrator at 4,000 ×*g*. Protein was then applied to a Cytiva HiLoad 16/600 Superdex 75 size exclusion column pre-equilibrated with SEC buffer (50 mM citrate-phosphate pH 8.0 or 8.5, 150 mM NaCl, and 10% glycerol). Fractions (2 mL) containing non aggregated protein were collected and concentrated to ~200 μL using 10 kDa spin concentrators.

CamB1

Cell pellets containing expressed CamB1 were resuspended in lysis buffer (4 mL/g wet cell pellet, 20 mM Tris Base, pH 8, 500 mM NaCl, 10% glycerol) and lysed using a Branson 150 Sonifier Cell Disruptor (70% amplitude, 10 total minutes, 15 sec on/45 sec rest). Lysate was cleared by centrifugation at 30,000 x*g* (20 min spin, 4°C). The soluble fraction was discarded, and the insoluble fraction resuspended in 60 mL of denaturing pH buffer: 8M urea, 100 mM Tris Base, 100 mM potassium phosphate dibasic, pH 8. Following denaturation and solubilization, the resuspended insoluble fraction was cleared by centrifugation (15,000 x*g*, 20 min, 4°C), divided evenly, and loaded directly onto 3, 5 mL gravity Ni-NTA columns pre-equilibrated with denaturing pH buffer. Bound protein was then washed with three column volumes (CVs) of denaturing pH buffer, pH 8, followed by an additional three CVs of the same buffer at pH 6.3. CamB1 was then eluted using five CVs of denaturing pH buffer, pH 4.5.

The pH of the resulting elution fractions was immediately increased to ~ 7-7.5 (verified by pH strip) with the addition of denaturing pH buffer, pH 8, or by direct addition of a minimal volume of 6M NaOH. pH adjusted fractions were then loaded onto 3 separate 5 mL gravity Ni-NTA columns preequilibrated with denaturing Ni-IMAC buffer A (8 M urea, 20 mM Tris Base, pH 8, 500 mM NaCl, 10% (v/v) glycerol). Bound protein was initially washed with 3 CVs denaturing Ni-IMAC buffer A and then eluted stepwise using single column volume aliquots of Ni-IMAC buffer A supplemented with the following concentrations of imidazole: 20, 50, 100, 150, 200, and 250 mM. Elution of residually bound CamB1 was completed by passing 3 CVs of denaturing Ni-IMAC buffer B over the resin (8 M urea, 20 mM Tris Base, pH 8, 500 mM NaCl, 10% glycerol, 500 mM imidazole). Fractions containing purified CamB1 were identified by SDS-PAGE before proceeding with refolding. CamB1 was refolded analogously to ArbB2 with the following exceptions: all buffers were prepared using 50 mM CAPS, pH 10, and arginine instead of 50 mM Tris Base, pH 8, and arginine-HCl; dialysis steps were limited to 4 hours each. Likewise, removal of CamB1 aggregates and subsequent preparation of purified CamB1 for assays was carried out as described for ArbB2.

**Gel Filtration Calibration**

The Cytiva Gel Filtration LMW Calibration Kit (Cytiva, # 28403841) was used to generate the protein standard curve shown in **Fig. S3**. Lyophilized standards were resuspended in SEC buffer (50 mM citrate, 50 mM potassium phosphate, pH 8, 150 mM NaCl, 10% (v/v) glycerol) according to the manufacturer’s instructions. The standards chosen for calibration of our HiLoad 16/600 Superdex 75 pg column include: Conalbumin (M_r_ 75,000) 3 mg/mL, Ovalbumin (M_r_ 44,000) 4 mg/mL, Carbonic Anhydrase (M_r_ 29,000) 3 mg/mL, Ribonuclease A (M_r_ 13,700) 3 mg/mL, Aprotinin (M_r_ 6500) 3 mg/mL. This mixture was injected onto the column pre-equilibrated with SEC Buffer using an ÄKTA go™ fast protein liquid chromatography system (Cytiva). Elution times were extracted from the corresponding chromatogram and plotted as follows: log (molecular weight) versus the gel phase distribution coefficient (K_av_). This is defined as K_av_ = (V_e_-V_o_)/(V_t_-V_o_); where V_e_: analyte retention volume; V_o_: void volume; V_t_: total volume. The elution volume used to calculate the molecular weight of apo-ArbB2 was estimated from the most active fraction shown in **Fig. S2**. The same fractions were used for assays completed with CamB1.

**Metal Titrations**

Substrate Preparation

For all assays, approximately 2 mg of lyophilized peptide (ArbA2_20mer_-FLWGY or CamA1_20mer_-ILLY) was resuspended in 1 mL of 50 mM citrate-phosphate buffer, pH 8.0. Peptide concentrations were then calculated from 280 nm absorbance values measured with a NanoDrop 2000 Spectrophotometer using extinction coefficients of ε_280nm_ = 6,990 M^-1^ cm^-1^ and 1,280 M^-1^ cm^-1^ for ArbA2_20mer_-FLWGY and CamA1_20mer_-ILLY, respectively.

Copper(II) Titration

To determine the stoichiometry of copper binding during turnover, 150 μM peptide (ArbA2_20mer_-FLWGY for ArbB2 reactions and CamA1_20mer_-ILLY for CamB1 reactions), 500 μM reductant (GSH or AscH), and varying concentrations of CuSO_4_ ( 3, 6 ,15, 30, 150, 300, 750, 1000 μM) at pH 8.0 was added to 50 mM citrate/phosphate buffer (prepared with potassium phosphate, dibasic). The reaction was initiated with 3 μM apo-BpC or with 500 μM GSH when indicated. Each point was quenched with two volumes of 75% (v/v) acetonitrile, 0.1% (v/v) formic acid following a one-hour incubation at room temperature and subjected to UPLC-MS analysis.

Inhibition of Substrate Cyclization with ZnSO_4_ and AgNO_3_

To determine the inhibitory effects of Zn(II) and Ag(I) on CamB1 catalysis, the concentration of ZnSO_4_ and AgNO_3_ was systematically varied in relative amounts to 150 μM CuSO_4_ (1:1, 1:10, 1:20, 1:50, 1:100, 1:500), 150 μM CamA1_20mer_-ILLY, 500 μM GSH, 50 mM citrate/phosphate buffer pH 8.0. Sulfate salts were used, when possible, due to their minimal coordination to copper (particularly relative to chloride). AgNO_3_ was used in place of Ag_2_SO_4_ because the sulfate salt is poorly soluble in aqueous buffer. Reactions were initiated with 3 μM apo-CamB1 (or with 500 μM GSH when indicated) then left at room temperature for one hour before quenching with two volumes of 75% (v/v) acetonitrile, 0.1% (v/v) formic acid. All assays were completed in triplicate and subjected to UPLC-MS analysis.

**UHPLC-HRMS Method**

UHPLC-HRMS analyses were performed on a Waters ACQUITY LC system coupled to an LTQ Orbitrap Elite (Thermo Scientific) mass spectrometer operated in positive ion mode. For routine assay measurements, samples were analyzed using an Agilent 1100 Series LC system equipped with a binary pump, autosampler, thermostatted column compartment, and diode-array detector, coupled to an Agilent 6130 single-quadrupole mass spectrometer operated in positive ion mode. Source settings: capillary voltage 3.5 kV (ESI+) / 3.0 kV (ESI−), nebulizer 35 psi, drying gas 10 L min^–1^ N_2_ at 325°C, Fragmentor voltage 60 V. Compound separation was accomplished using the following solvents: solvent A, water with 0.1% (v/v) formic acid, and solvent B, acetonitrile with 0.1% (v/v) formic acid. The chromatography method was completed as follows: 15% B for 1 min (directed to waste), 15-30% B gradient for 11 min, 30-100% B for 2 min, and 100-15% B gradient for 1 min. The columns used for chromatographic separation were a Kinetex^®^ 1.7 μm C18 100 Å , 50 x 2.1 mm, ACQUITY UPLC^®^ BEH C18 1.7 μm 2.1 × 50 mm, and bioZen™ 1.7 μm Peptide XB-C18 2.1 × 50 mm column. Data was processed in MestReNova v16.0 (MSChrom plugin), mass tolerance of 10 ppm, and mass precision of four decimals. Conversion of linear peptide to the cyclized product was quantified using absorbance traces at 195 or 280 nm (for CamA1_20mer_-ILLY and ArbA2_20mer_-FLWGY, respectively) or, more frequently, using extracted-ion chromatograms (XICs) targeted to the most abundant m/z values of the substrate and product with a narrow tolerance (± 10-50 ppm) (**Table S3, Fig. S4**). For each species, we restricted integration to a single adduct/charge state and applied baseline-corrected area-under-the-curve (AUC) integration within retention-time windows defined by local minimum between the partially co-eluting peaks. These settings, along with fixed RT windows applied consistently across replicates, minimized cross-assignment of shared signal.

**Peptide Mass Fingerprinting (PMF) by In-gel Digestion and LC-MS/MS**

Gel bands were excised and destained, then treated with 10 mM dithiothreitol (DTT) and 50 mM iodoacetamide to reduce disulfide bonds and alkylate cysteine residues. Proteins were digested overnight at 37°C with 500 ng sequencing-grade trypsin (Thermo).^7^ The resulting peptides were analyzed by ESI-MS/MS on an Orbitrap Fusion Lumos mass spectrometer (Thermo). Peptide samples were loaded onto a 50 cm μPAC analytical column (PharmaFluidics) using an Easy-nLC 1200 UPLC/autosampler (Thermo). Chromatography was performed at a flow rate of 500 nL/min with a 90 min gradient from 2% to 45% solvent B in solvent A. Solvent A consisted of 2:98 acetonitrile: water with 0.1% (v/v) formic acid, and solvent B consisted of 80:20 acetonitrile: water with 0.1% (v/v) formic acid. For electrospray ionization, a μPAC-compatible Easy-Spray emitter (PharmaFluidics) was operated at 1700 V. The ion transfer tube temperature was 275 °C, RF lens was set to 30%, and the default charge state was 3. MS scans were acquired over m/z 400-1500 in positive profile mode at 120,000 resolution with an AGC target of 4.0 × 10^5^ and a maximum injection time of 50 ms. Precursor ions selected for MS/MS met the following criteria: isotopic distribution consistent with peptides, intensity above 2.0 × 10^4^, and charge states between 2 and 5. Dynamic exclusion was applied for 15 s after a single selection, with MS/MS acquisition triggered at 45% of chromatographic peak width (expected FWHM: 15 s). MS/MS spectra were collected starting from m/z 150 at 15,000 resolution in positive centroid mode, using an AGC target of 1.0 × 10^5^ and a maximum injection time of 200 ms. Higher-energy collisional dissociation (HCD) was performed with stepped collision energies of 25-35%. Mascot Distiller was used to generate Mascot Generic Format (MGF) peak lists, which were searched with an in-house Mascot Server against the *E. coli* database appended with the pelB-His_6_-CamB1 amino acid sequence. Moreover, Protein identification was confirmed and visualized with MetaMorpheus (GUI, version 1.1.4) using the standard Calibration task followed by the Search task. Thermo RAW files were analyzed directly; when conversion was required, vendor files were centroided to mzML with ProteoWizard msConvert (version 3.0.25204-2ad5a87).^8^ Searches were performed against a single FASTA comprising the *E. coli* host proteome (strain matching the expression system), the CamB1 construct sequence including any affinity tags or linkers, and a small contaminants panel (trypsin, keratins, BSA/caseins, and relevant tag/resin proteins). MetaMorpheus^9^ generated decoys automatically and performed target-decoy competition. (**Figs. S6-S8**). Although the CamB1 gel-band assignments show only 20-25% sequence coverage, protein identity did not rely on coverage alone. Instead, we required orthogonal, quantitatively stringent evidence. (i) **Gel consistency:** The apparent SDS–PAGE molecular weight matched the theoretical mass of CamB1 (including tags/linkers) within the typical gel error window (~5-10%), and no higher-abundance co-migrants were supported at comparable confidence. (ii) **Quantitative MS fit:** High-confidence PSMs showed ppm-level precursor mass errors and coherent b/y-ion series with informative consecutive ions and low fragment-ion mass errors, reproduced across technical replicates. (iii) **Controlled error rate:** Target–decoy competition was used to control false discoveries, with identifications filtered at 1% FDR at both the PSM and peptide levels; protein inference used parsimony and was reported at 1% protein-level FDR. We further required **proteotypic uniqueness**: at least two peptides unique to CamB1 among all sequences in the searched database (or, in rare cases, a single unique peptide whose MS/MS spectrum exhibited near-complete b/y coverage, very low mass error, and a high search-engine score, e.g., a Mascot-type ion score). Together, these criteria provide statistically controlled, instrument-calibrated evidence for CamB1 identity even when overall sequence coverage is modest. Table of b- and y-ions used in the fingerprinting analysis of the two bands in **Figs. S6-S10** are in **Table S4** and **Table S5**.

**DNA, Protein, and Peptide Sequences with Accession Numbers**

>ArbB2 – BURP cyclase protein [*Coffea arabica*] XP_027066250.1

MTCKGNNTYQLMDSKALASCVLLLHLLIVLGACDIIPKAKYSGTNAIRLHSMDANNPHRNDKTHHVAHVHEKKSMHDPSLSSSHMMHQIDPRATVFFVLDDLKLGKTLSILFPDGDPSPLSSPYLWPREQADAIPFSLAKLPQILQHFSFPQGSRKAQVMEHALRACETKPMKGEPKACATSYESLVDFARKILGLNTDIEVLSTHRLTKSNAARLQNYTITEAPERISTLKMVGCHTMPYPYIVFYCHYQQGDNRLYRTVLSGENGDRVEGLAICHMDTSQWNHDHVSFQVLGIEPGTAPVCHFFPAEDFVLVPSTSSI

>ArbB2 – codon optimized sequence for expression in *E. coli*

ATGCACCATCATCATCATCATTTGGTGCCGCGTGGTTCTGTTCTCCTTCTGCATTTACTCATTGTGTTGGGCGCCTGTGATATCATTCCGAAGGCAAAGTATAGTGGGACAAACGCTATTCGATTGCATAGTATGGATGCTAACAATCCTCATCGTAACGACAAAACCCATCACGTGGCACACGTACATGAAAAGAAGTCAATGCATGATCCGTCTCTGTCCAGCTCTCATATGATGCACCAAATCGACCCTCGTGCAACCGTCTTTTTTGTTTTAGACGATCTGAAGTTAGGTAAAACACTGAGCATCCTCTTTCCGGATGGCGACCCGTCCCCGTTGTCTTCCCCATATCTGTGGCCTCGTGAACAAGCTGATGCGATCCCCTTCAGCCTGGCAAAGCTGCCTCAGATTCTGCAGCATTTTTCGTTTCCGCAGGGTTCACGTAAGGCACAAGTGATGGAACACGCGCTTCGAGCCTGTGAAACTAAACCCATGAAAGGTGAACCGAAAGCTTGTGCCACTTCCTACGAATCGTTGGTTGATTTTGCTCGGAAGATACTGGGCCTCAACACAGATATTGAGGTCCTGAGCACACATCGGCTTACTAAAAGTAATGCCGCACGATTACAGAACTATACCATAACGGAAGCGCCAGAGCGTATCTCAACCCTGAAAATGGTAGGTTGTCATACGATGCCGTATCCATATATCGTGTTCTATTGCCATTATCAACAGGGCGATAATCGTCTGTATCGCACCGTGTTATCAGGAGAGAATGGTGATCGCGTGGAAGGCCTTGCTATATGTCACATGGATACCTCTCAATGGAATCATGATCATGTCAGCTTTCAGGTGTTGGGTATCGAGCCGGGAACTGCTCCGGTTTGCCATTTTTTTCCAGCTGAAGACTTCGTTCTGGTACCGTCGACGTCCTCTATA

>His_6_-ArbB2ΔV21 (truncated expressed protein in *E. coli* with thrombin-cleavable 6XHis-tag)

HHHHHHLVPRGSVLLLHLLIVLGACDIIPKAKYSGTNAIRLHSMDANNPHRNDKTHHVAHVHEKKSMHDPSLSSSHMMHQIDPRATVFFVLDDLKLGKTLSILFPDGDPSPLSSPYLWPREQADAIPFSLAKLPQILQHFSFPQGSRKAQVMEHALRACETKPMKGEPKACATSYESLVDFARKILGLNTDIEVLSTHRLTKSNAARLQNYTITEAPERISTLKMVGCHTMPYPYIVFYCHYQQGDNRLYRTVLSGENGDRVEGLAICHMDTSQWNHDHVSFQVLGIEPGTAPVCHFFPAEDFVLVPSTSSI

>ArbA2 – precursor peptide [Coffea arabica] XP_027066141.1

MASSITLIAVFSIALFACITEARKNPTDFLQSAVINEHTEDNHHAESSLSNQKKTSNGNTLKDFESKPGSFLWGYQGNDAESKSKEEKPLMKGFESKPGSFLWGYQGNDAESKSKEEKPLMKGFESK PGSFLWGYQGNDVESKSKEEKPLMKDFESKPGSFL

>ArbA2_20mer_-FLWGY

GNTLKDFESKPGSFLWGYQG

>Extended-ArbA2_20mer_-FLWGY

MRKNPTDFLQSAVINEHTEDNHHAESLSNQKKTSNGNTLKDFESKPGSFLWGYQGDAESKSKEEKPLMKGFESKPGSFL

>CamB1 (*Ceanothus americanus*, see reference 4)

MAKGFASCVLILYLFFLMCSSGNGSDHQESANIVENHSHAHNNPMSHNMDYDMYMAPRVGFFTGDDLHVGKTMTVQFFTKDPSSLPPFLSREEADRIPFSLNEFPHLLKLFSFSQGSHEAKLIERTLQTCAQKPIVGERKTCATSKESLVEFVSSVLGGRNGVDFRALKSTHLGKPSSTFQNYTFLDVKEVNSPNMVACHIMDYPYAVFVCHTQTSKVYQILLAGHEDGDIINAVAVCHIDTSHWAPDHISFRFLGVKPGTVPVCHFFGPHNLIWVQN

>CamB1 (codon optimized for expression in *E. coli*)

ATGAAATACCTGCTGCCGACCGCTGCTGCTGGTCTGCTGCTCCTCGCTGCCCAGCCGGCGATGGCCATGCATCACCATCATCACCATGAAAATCTGTACTTCCAGTCTAATGGTAGCGATCATCAGGAGAGTGCTAATATTGTCGAAAACCATAGTCACGCGCACAATAATCCGATGTCTCATAACATGGATTATGACATGTACATGGCACCCCGGGTAGGCTTCTTTACGGGAGACGACCTTCATGTTGGCAAAACAATGACCGTTCAGTTCTTTACAAAGGATCCGTCCAGTTTGCCCCCATTTCTGTCGCGAGAAGAGGCAGACCGCATTCCGTTTAGTTTAAATGAATTTCCGCACCTGCTTAAGCTGTTTTCTTTTAGCCAGGGATCCCATGAAGCTAAGTTGATCGAACGTACACTCCAGACCTGTGCCCAAAAGCCCATTGTTGGGGAGCGTAAAACCTGTGCGACAAGTAAAGAATCCCTGGTGGAGTTTGTGTCTTCTGTCCTGGGTGGTCGTAATGGCGTGGATTTTCGCGCACTGAAATCGACCCATCTGGGCAAACCGTCTTCAACGTTCCAGAACTACACCTTTCTGGACGTTAAAGAGGTCAACTCCCCCAATATGGTCGCATGCCACATAATGGATTACCCATATGCTGTTTTCGTATGTCATACTCAAACTAGCAAGGTGTACCAGATCCTGCTGGCCGGGCATGAAGATGGTGATATAATCAACGCTGTCGCCGTTTGTCATATAGACACGTCACATTGGGCTCCAGATCACATTTCTTTCCGCTTTCTTGGAGTAAAACCAGGGACAGTTCCCGTATGTCATTTCTTCGGGCCGCATAATCTTATCTGGGTTCAAAACTAG

>pelB-His_6_-CamB1 (truncated expressed protein in *E. coli* with TEV-cleavable 6XHis-tag)

MKYLLPTAAAGLLLLAAQPAMAMHHHHHHENLYFQSNGSDHQESANIVENHSHAHNNPMSHNMDYDMYMAPRVGFFTGDDLHVGKTMTVQFFTKDPSSLPPFLSREEADRIPFSLNEFPHLLKLFSFSQGSHEAKLIERTLQTCAQKPIVGERKTCATSKESLVEFVSSVLGGRNGVDFRALKSTHLGKPSSTFQNYTFLDVKEVNSPNMVACHIMDYPYAVFVCHTQTSKVYQILLAGHEDGDIINAVAVCHIDTSHWAPDHISFRFLGVKPGTVPVCHFFGPHNLIWVQN

>His_6_-CamB1

HHHHHHENLYFQSNGSDHQESANIVENHSHAHNNPMSHNMDYDMYMAPRVGFFTGDDLHVGKTMTVQFFTKDPSSLPPFLSREEADRIPFSLNEFPHLLKLFSFSQGSHEAKLIERTLQTCAQKPIVGERKTCATSKESLVEFVSSVLGGRNGVDFRALKSTHLGKPSSTFQNYTFLDVKEVNSPNMVACHIMDYPYAVFVCHTQTSKVYQILLAGHEDGDIINAVAVCHIDTSHWAPDHISFRFLGVKPGTVPVCHFFGPHNLIWVQN

>CamA1 (see reference 4)

GNFFFYHNNKNAVAKSKADEGELKDFSVDPSGNFFFYHNNKNAVAKSKADEGELKDFSVDPSGNFFFYHNNKNAVAKSKADEGELKDFSVDPSGNFFFYHNNKNAVAKSKADEGELKDFSVDPSGNILLYHGNKNAEVKSNTDGKHKEDFSVDPSGNPLLYHDNKNDDFSVDPSGNPLLYHDNKNGEHKDGFSVDPSGNPLFYHGNKNDDFSVDPSGNPLEYHGNKNGEHKDGFSVDPSGNPLLYHDNKNDDFSVDPSGNPLEYHGNKNGEHKDGFSVDPSGNPLFYHGNKNGEHKDGFSVDPSG

>CamA1_20mer_-ILLY

EGELKDFSVDPSGNILLYHG

>Extended-CamA1_20mer_-ILLY

KNAVAKSKADEGELKDFSVDPSGNILLYHGNKNAEVKSNTDGKHKEDFSVDPSGNPLLYHDNKNDDFSVDPSGNPLLYHDNKNGE

**Supplemental Figures**

**Fig. S1.** 12% SDS-PAGE analysis of ArbB2 whole cell expression over time: Lanes 1 & 10, molecular weight markers (with the molecular weight highlighted beside the bands); Lanes 2-9 represent whole cell expression samples (1 mL) standardized by their OD_600nm_.


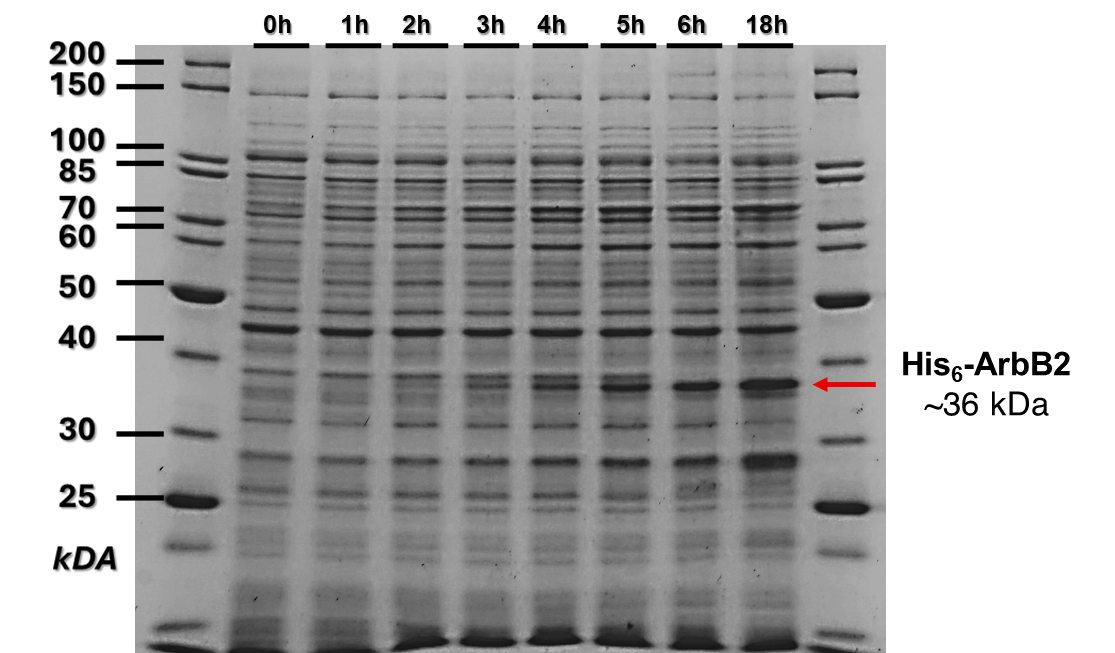


**Fig. S2**. (**A**) Size exclusion chromatography of apo-ArbB2 (HiLoad 16/600 Superdex 75) after purification and refolding through dialysis (black trace). Enzyme activity of each 2 mL fraction with 5 μM Apo-ArbB2, 1 mM CuSO_4_, 500 μM GSH, 150 μM ArbA2_20mer_-FLWGY, 1 hour (Green dotted line). (**B**) 12% SDS-PAGE gel of each of the relevant 2 mL fractions collected to determine the relative contribution of apo-ArbB2 to the chromatograph (numbers above each gel lane correspond to the fraction number in (**A**). The left most lanes of each gel are the standard ladder, and the loading fraction is denoted as *LO*.


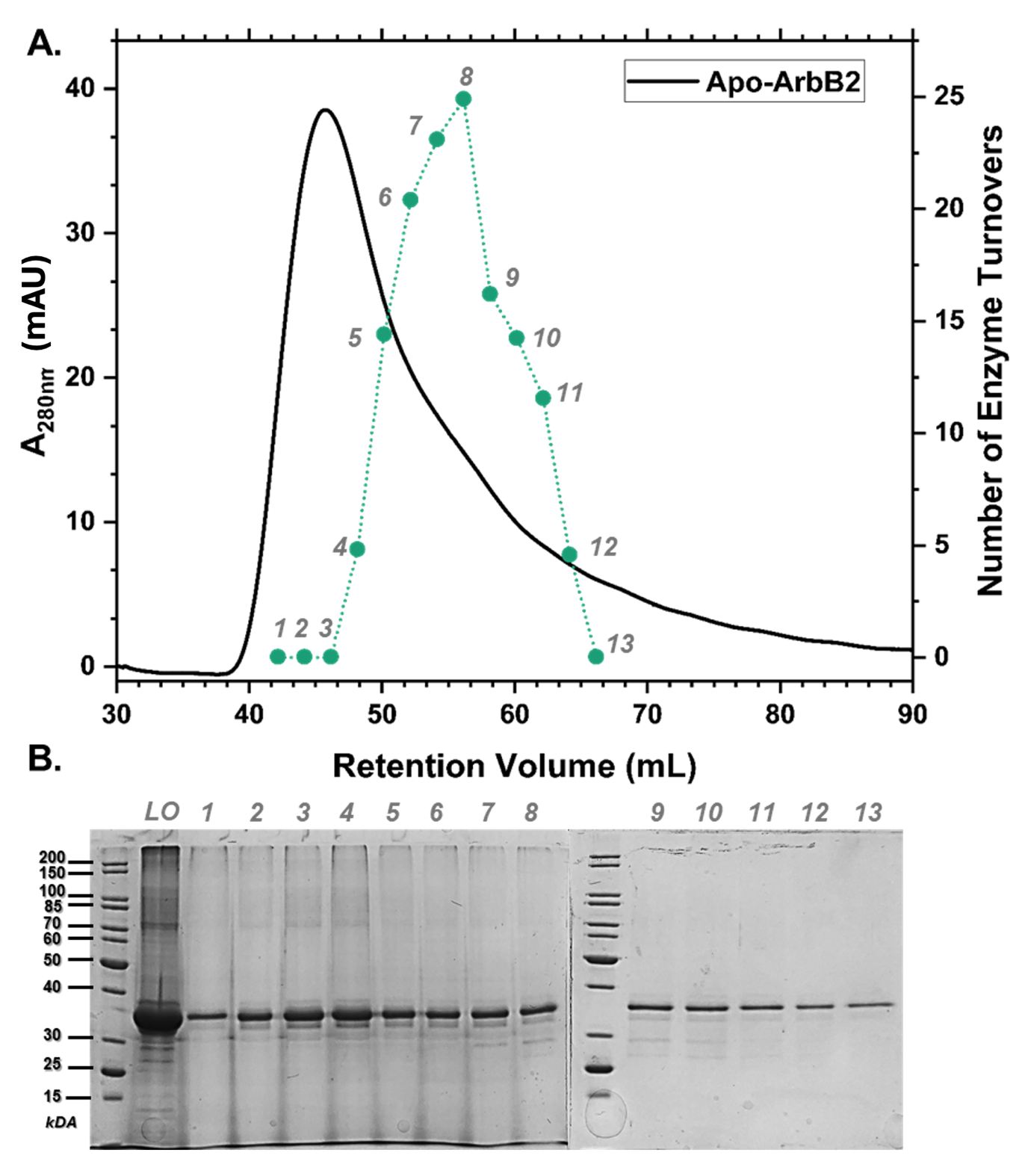


**Fig. S3**. Size exclusion chromatogram of standards (Conalbumin, 75,000 Da, Ovalbumin, 44,000 Da, Carbonic Anhydrase, 29,000 Da, Ribonuclease A, 13,700 Da, and Aprotinin, 6,500 Da) loaded onto a HiLoad 16/600 Superdex S75 column (solid black line). Individual peak fitting results (dashed gray lines), generated using the Multipeak Fitting package in Igor Pro 9 (WaveMetrics), were used to obtain the elution volume of each standard, with the sum of all individual peaks shown in red. *Inset*: K_av_ vs log(MW) calibration plot derived from the elution volumes of each standard. The molecular weight of ArbB2 calculated from this plot is shown in red.


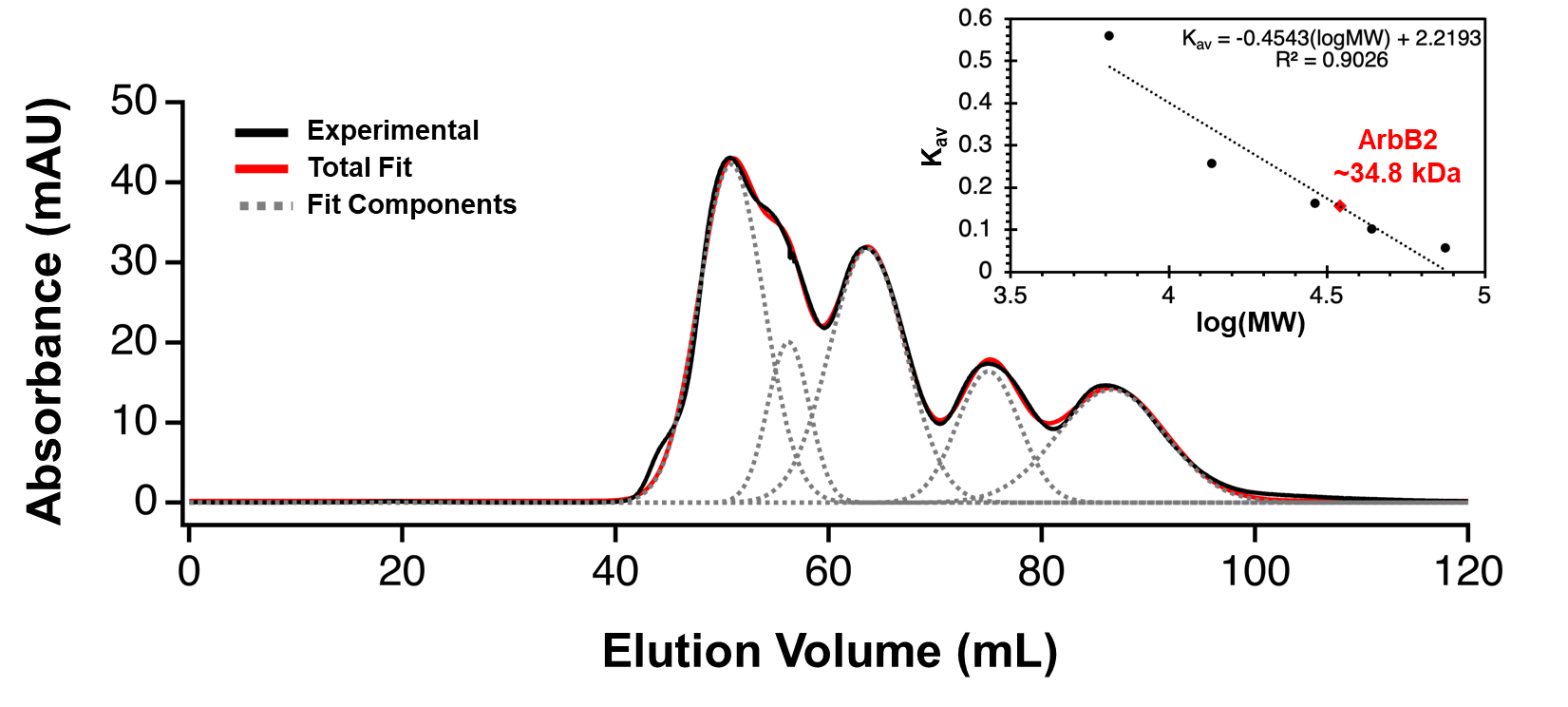


**Fig. S4.** (**A**) Total ion chromatograms of ArbA2_20mer_-FLWGY before and after cyclization (**B**) Example extracted ion chromatograms of the substrate (744.69 ± 10 ppm) and product (744.03 ± 10 ppm) used to determine the area under the curve for tracking reaction progression (percent conversion). (**C**) MS^1^ signals of (top) linear ArbA2_20mer_-FLWGY most abundant m/z, and (bottom) its corresponding cyclized product (*z* = 3). Green vertical dotted lines represent the theoretical isotope envelopes.


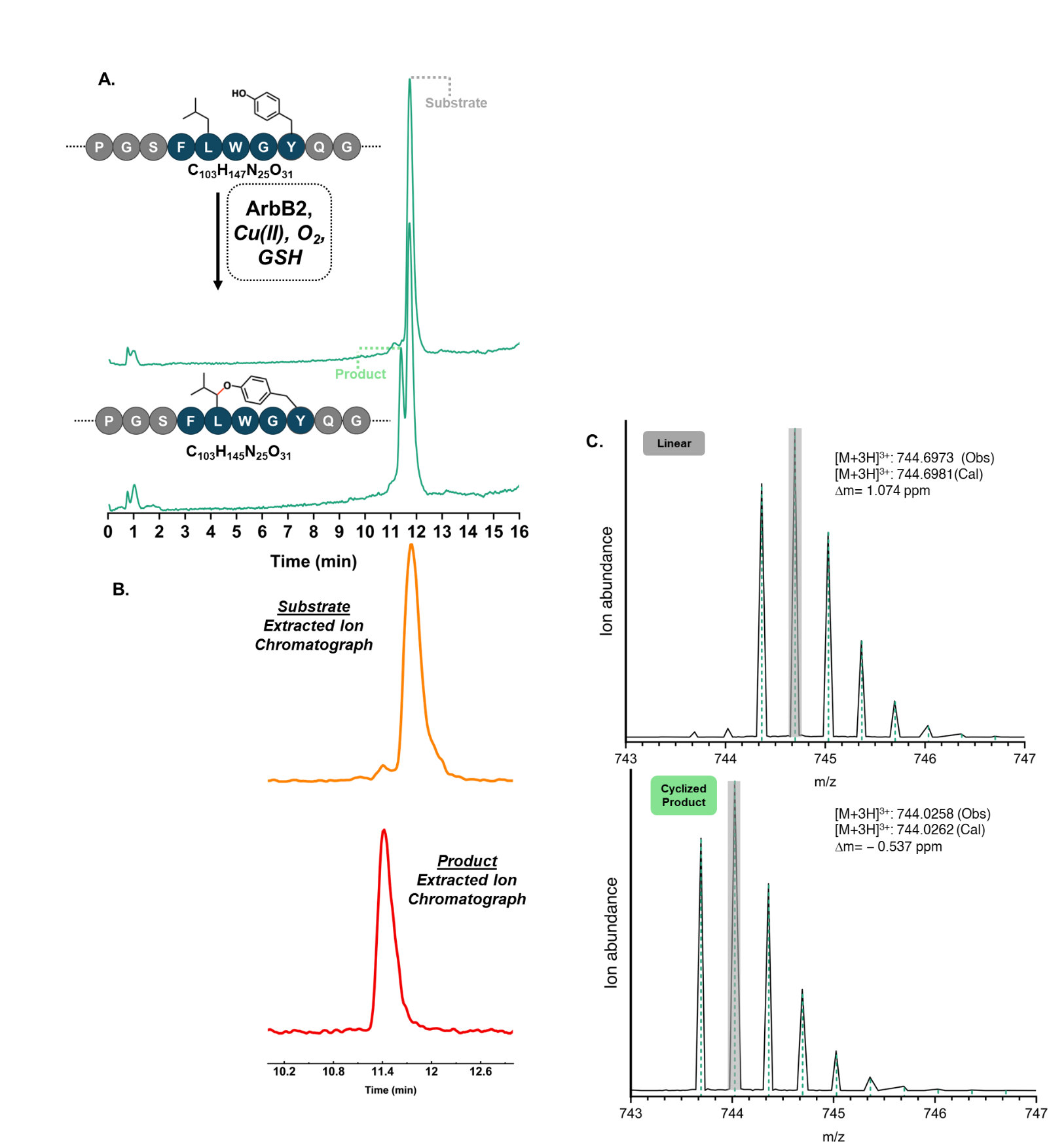


**Fig. S5.** 12% SDS-PAGE analysis of (**A**) Co-purified impurity (**CamB1’**) with **CamB1**. Lane 1 represents the molecular weight markers (with the molecular weight highlighted beside the bands), Lane 3: Soluble fraction after the final concentrating step, Lane 6: Precipitated fraction after concentrating. (**B**) Lane 6 represents **His_6_-tag-free CamB1** after TEV cleavage/purification. *Lanes labeled X are empty or irrelevant.*


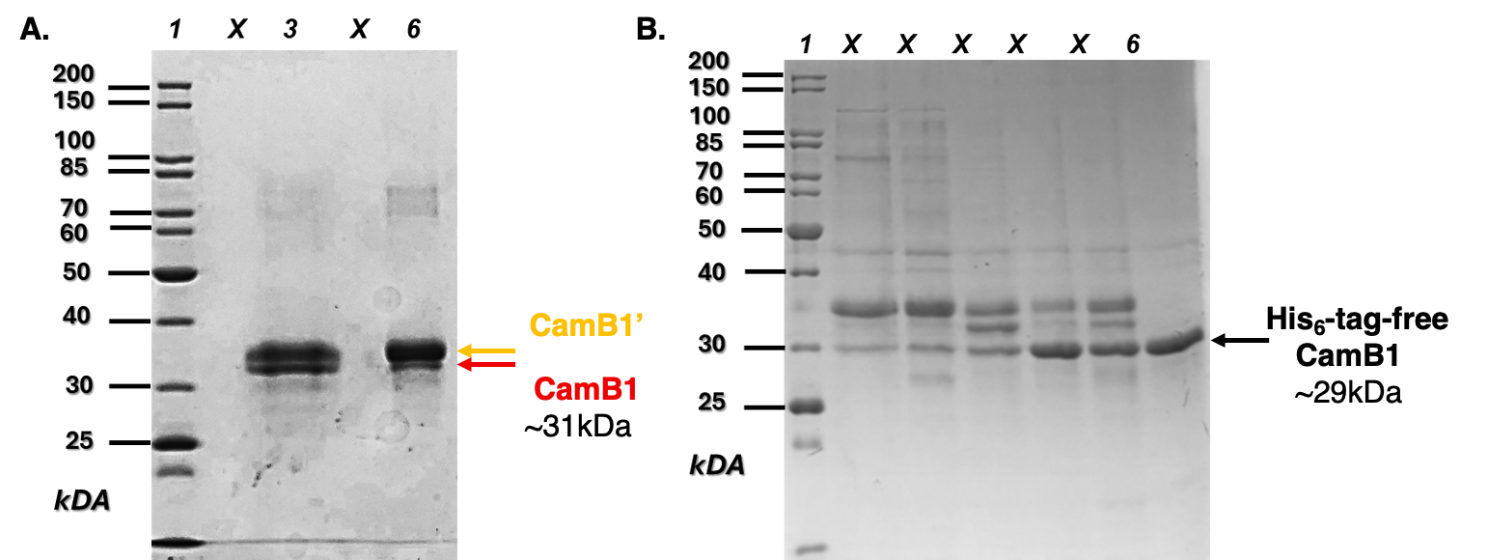


**Fig. S6.** In-gel tryptic digestion of CamB1: (**A**) Coomassie-stained SDS–PAGE gel showing the target protein bands (Band 1 and 2) excised for in-gel tryptic digestion analysis (same gel as **Fig. S5**). Extracted peptides were analyzed by LC–MS/MS on an Orbitrap mass spectrometer, and representative MS^1^ spectrum for Band 1 (**B**) and Band 2 (**C**) along with pelB-His_6_-CamB1sequence coverage of matched peptides obtained from the MS^2^ b- and y-ion fingerprinting experiment. Protein sequence coverage was calculated to be 20% and 25% for band 1 and band 2, respectively. (Bands labeled with a red X were carefully excised around to not include it in the digestion process).


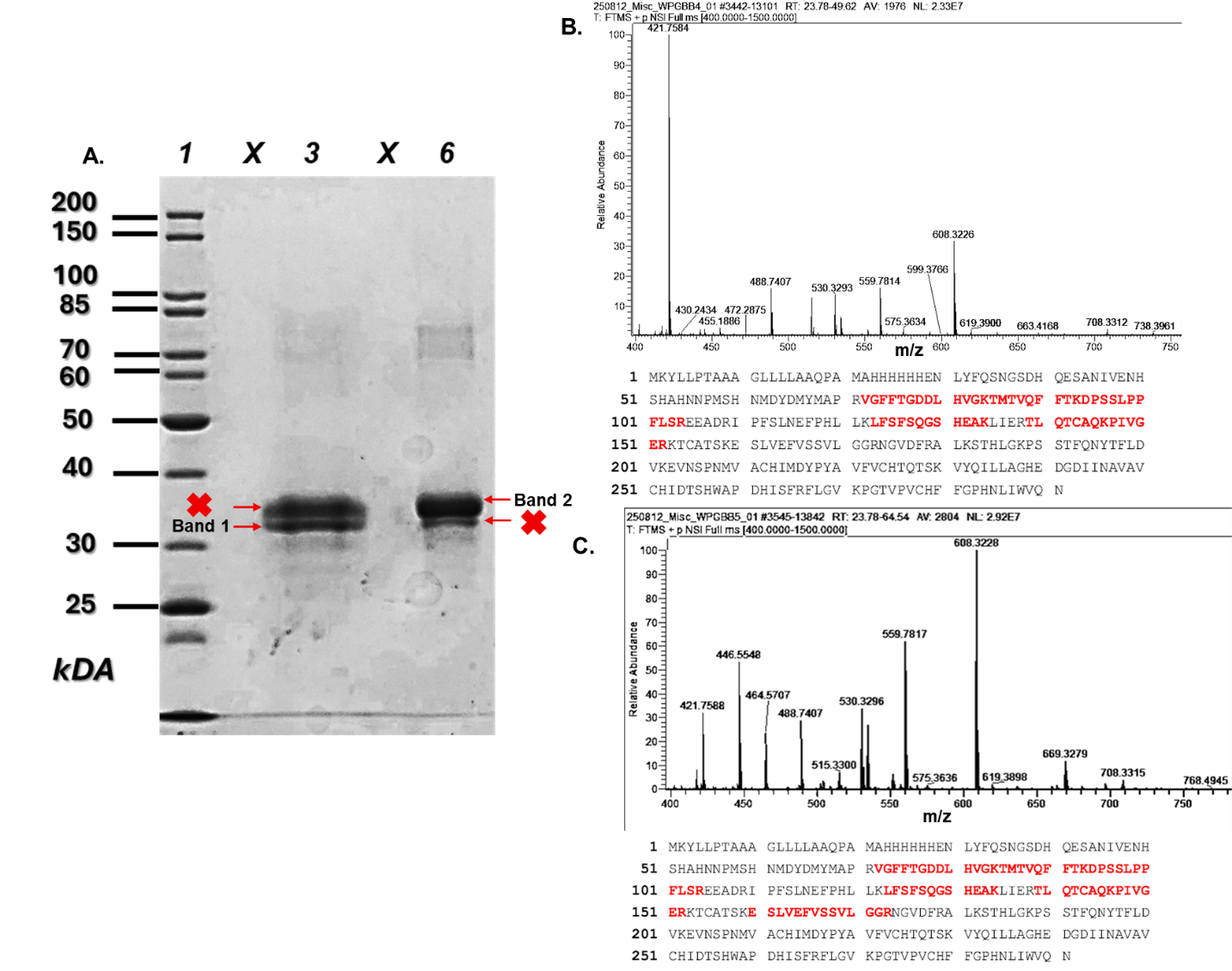


**Fig. S7.** Example MS/MS spectrum of the doubly charged precursor ion at 608.32 m/z from Band 1, corresponding to the tryptic peptide sequence DPSSLPPFLSR. Prominent y-ions are annotated with sub-ppm mass accuracy, confirming peptide sequence assignment. All b- and y-ions analyzed in fingerprinting analysis can be found in **Table S4**.


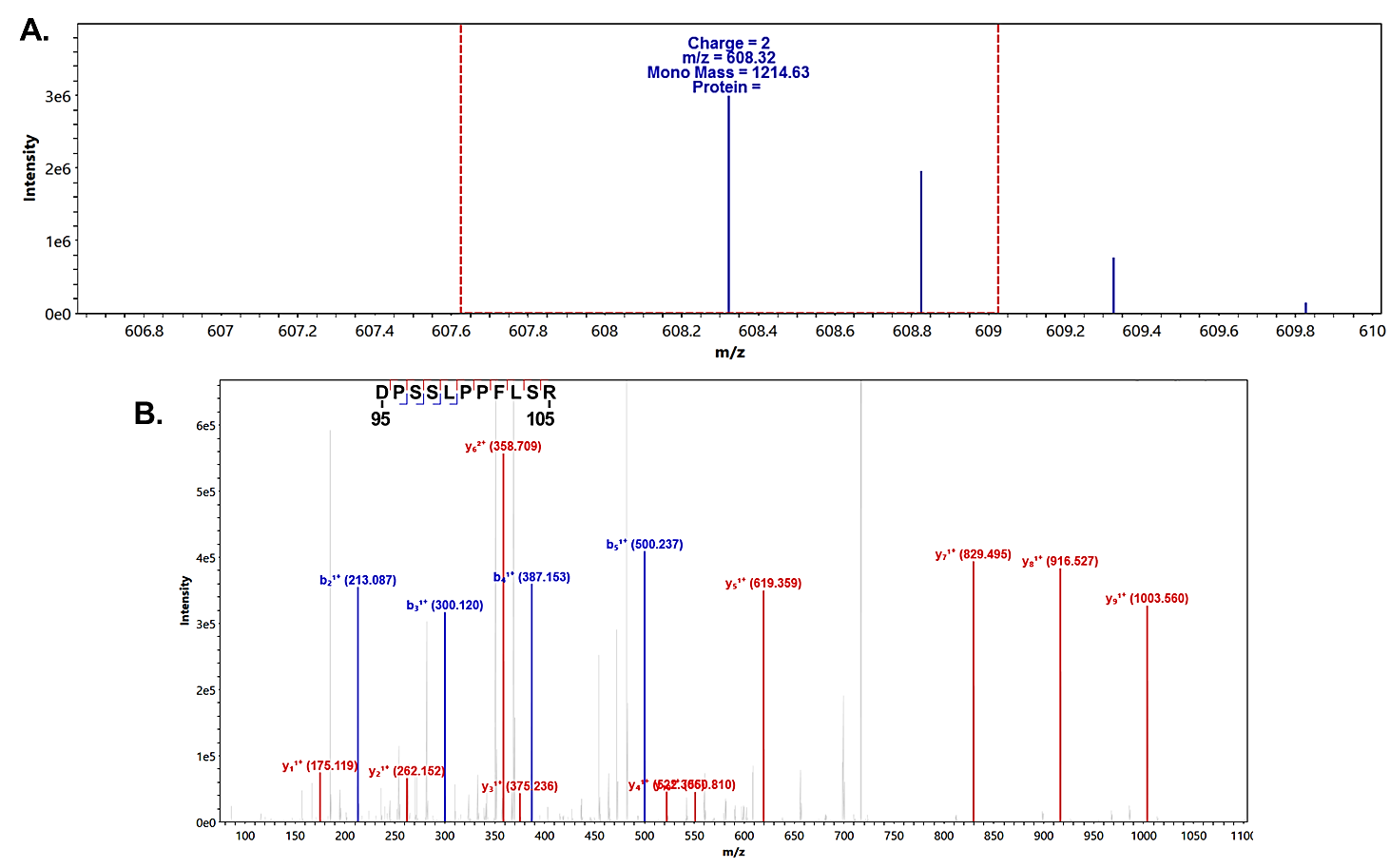

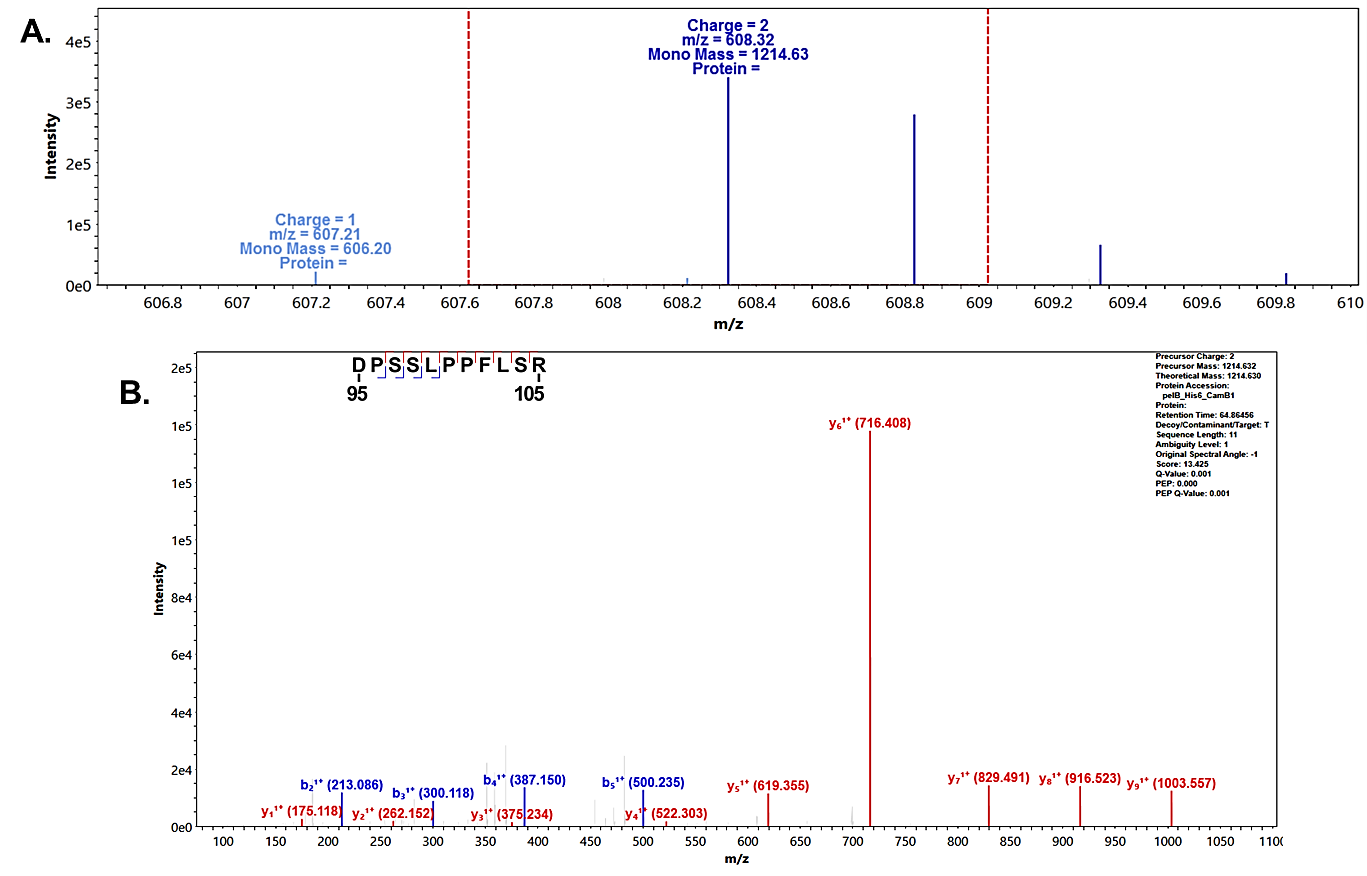


**Fig. S8.** Example MS/MS spectrum of the doubly charged precursor ion at 608.32 m/z from Band 2, corresponding to the tryptic peptide sequence DPSSLPPFLSR. Prominent y-ions are annotated with sub-ppm mass accuracy, confirming peptide sequence assignment. All b- and y-ions analyzed in fingerprinting analysis can be found in **Table S5**.

**Fig. S9.** Total ion chromatograms of CamA1_20mer_-ILLY (**A**) before and (**B**) after cyclization by CamB1. (**C**) and (**D**): MS^1^ signals of linear CamA1_20mer_-ILLY most abundant m/z, and its corresponding cyclized product (*z* = 3). Green vertical dotted lines represent the theoretical isotope envelopes.


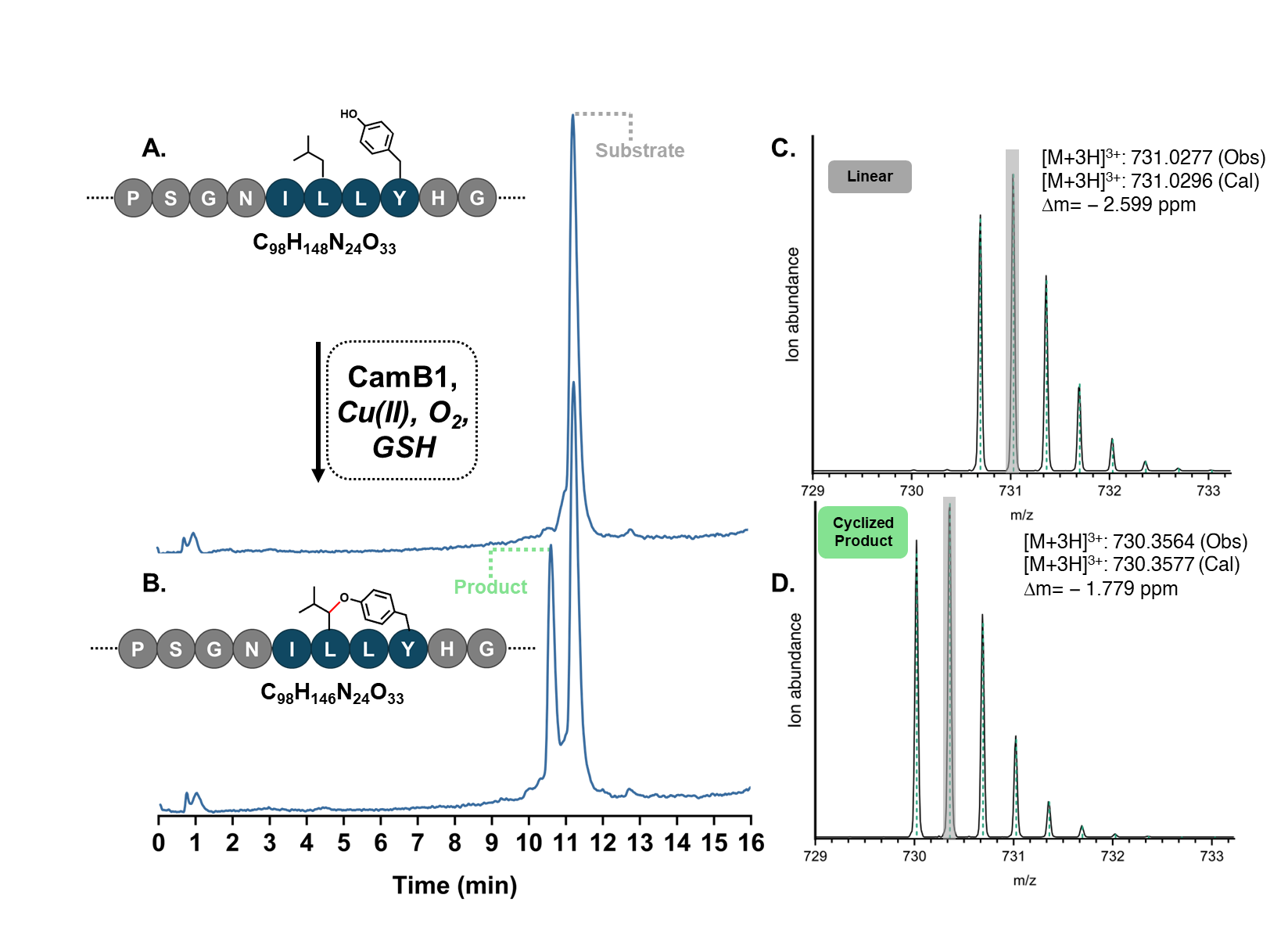


**
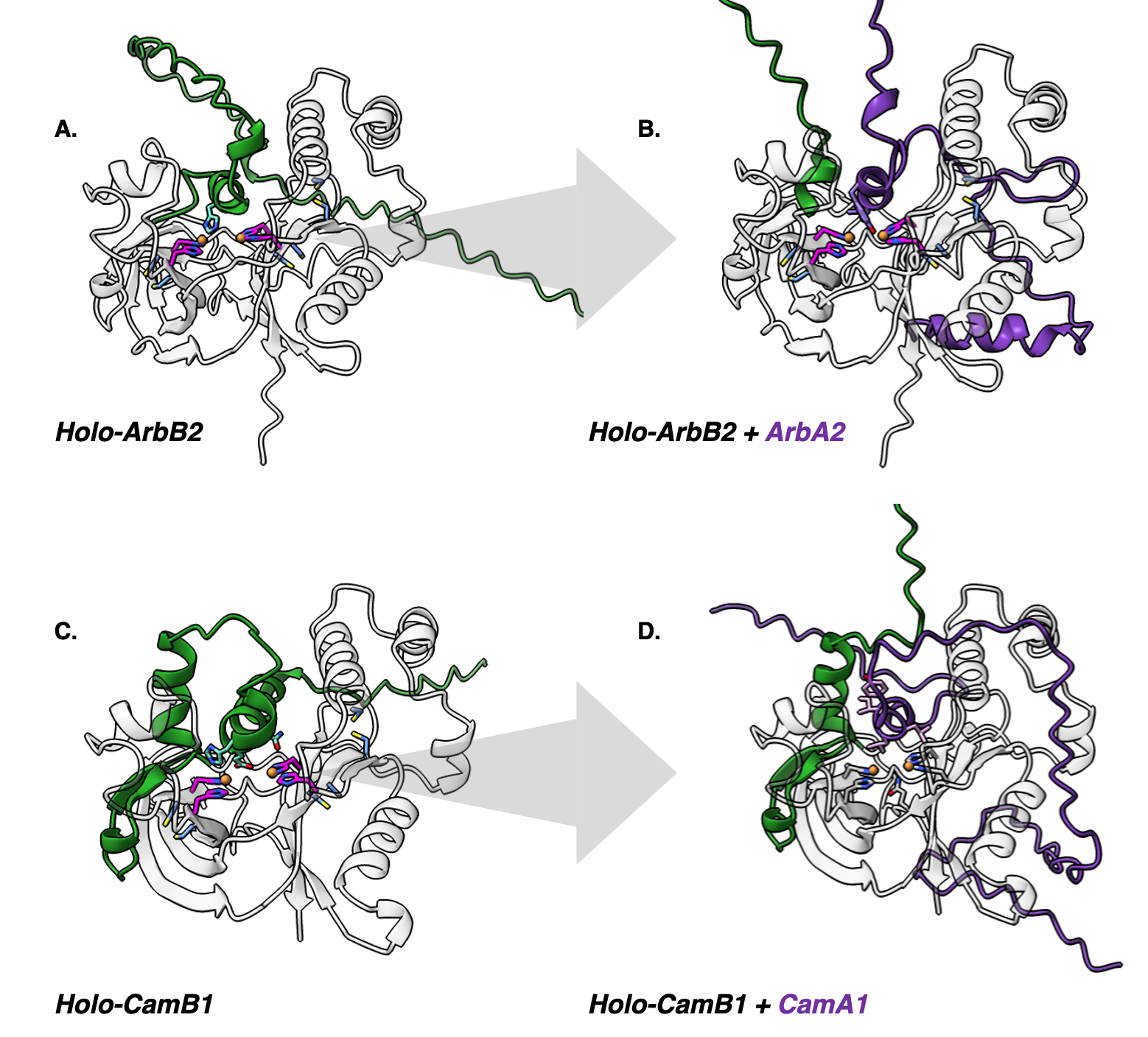
**

**Fig. S10.** AlphaFold 3 models of holo-ArbB2 (gray) in the (**A**) absence and (**B**) presence of the peptide substrate extended-ArbA2_20mer_-FLWGY (purple). Analogous models of holo-CamB1 (gray) shown in (**C**) and (**D**) depict the protein in the absence and presence of the peptide substrate extended-CamA1_20mer_-ILLY (purple), respectively. For clarity, in all models, the respective N-termini of BpCs are colored green. In general, these models predict the displacement of the N-terminus of both ArbB2 and CamB1 from the active site in response to substrate binding.

**
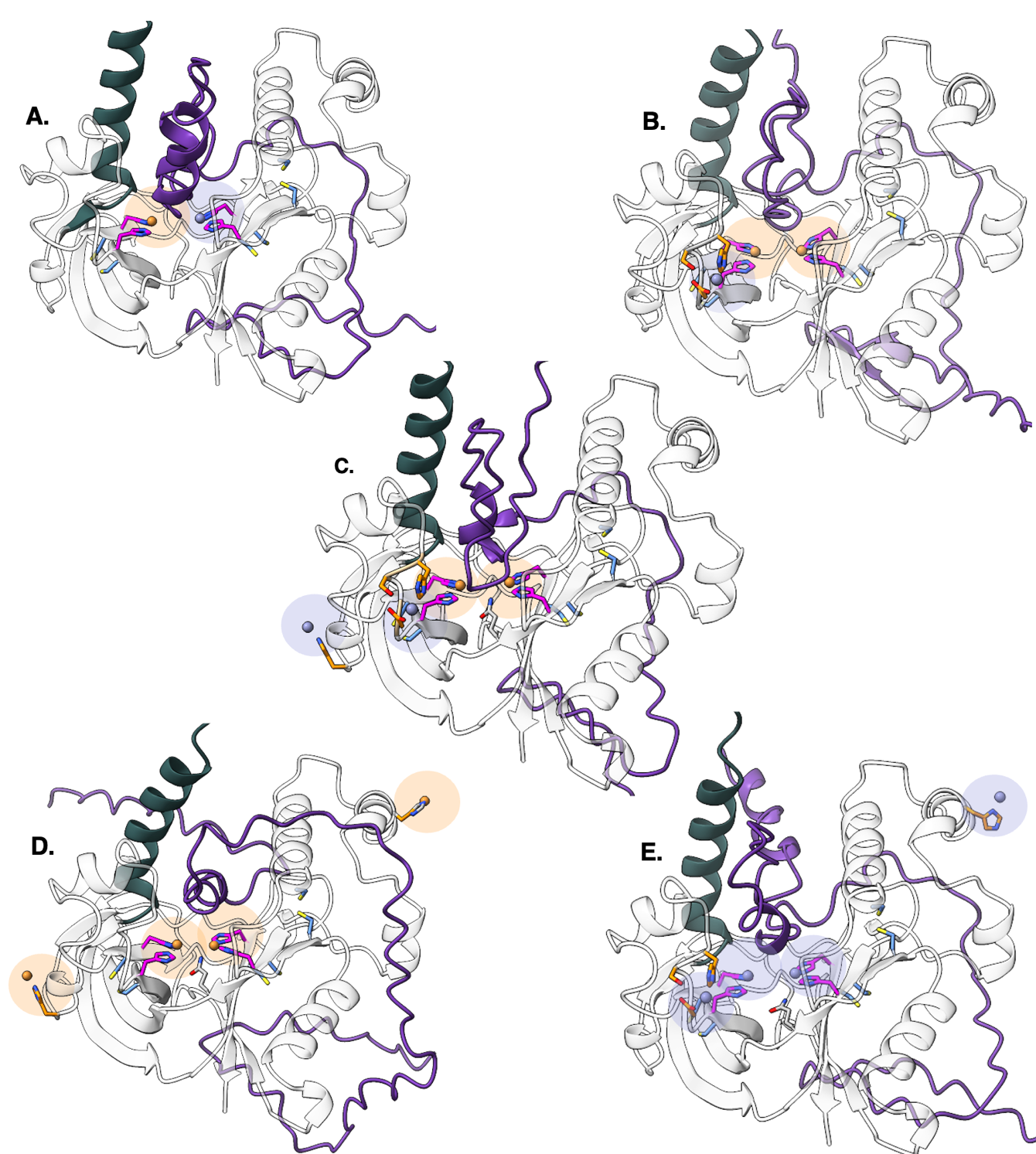
**

**Fig. S11.** AlphaFold 3 models of holo-CamB1 (gray) bound to the peptide substrate extended-CamA1_20mer_-ILLY (purple) with different stoichiometries of copper (orange spheres) and zinc (purple spheres). (**A**) 1 Cu, 1 Zn; (**B**) 2 Cu, 1 Zn; (**C**) 2 Cu, 2 Zn; (**D**) 4 Cu; and (**E**) 4 Zn. For clarity, in all models, the N-terminus of CamB1 is colored green. Any residues predicted to coordinate metal ions outside of the active site are shown in orange.

**Supplemental Tables**

**Table S1. Constructs used for recombinant expression of BpCs in *Escherichia coli*.**

|  | **Protein (Organism)** | | **Associated Burpitide** | | **Expression Vector** | **Vector Description** | | **N-terminal Truncations** | | **Main Text Reference(s)** | |  |
| --- | --- | --- | --- | --- | --- | --- | --- | --- | --- | --- | --- | --- |
| Autocatalytic (Fused) Systems | SkrBURP (*Selaginella kraussiana*) | | selanine A/B | | pVP57K | pVP56K derivative encoding an N-terminal MBP-fusion protein with 8X-His tag | | Residues M1-A20 removed (signal peptide); residues A60-D266 removed in favor of single core | | 18 | |  |
|  | CcaBURP1 (*Cercis canadensis*) | | cercic acid | |  |  |  | Residues M1-A22 removed (signal peptide); residues R68-W461 removed in favor of single core | | 18 | |  |
|  | CcaBURP2 (*Cercis canadensis*) | | stephanotic acid-(LV) | |  |  |  | Residues M1-A21 removed (signal peptide); residues Q67-W227 removed in favor of single core | | 18 | |  |
|  | KjaBURP (*Kerria japonica*) | | moroidin | |  |  |  | Residues M1-A21 removed (signal peptide); residues V83-H140 removed in favor of single core | | 27 | |  |
|  | AhyBURP (*Arachis hypogaea*) | Legumenin / lyciumin I | | pHis_8_ | | | pET28 derivative with N-terminal 8XHis-tag | | Residues M1-G21 removed (signal peptide) | | 18, 24 | |
| Split Systems | ArbB2 (*Coffea arabica*) | arabipeptin A | | pET28-MBP | | | N-terminal MBP-fusion protein with 6XHis-tag | | Residues M1-C33 removed (signal peptide); residues D34-S74 removed | | 23 | |
|  | ArbB3 (*Coffea arabica*) | arabipeptin A | | pET28-MBP | | |  |  | Residues M1-C22 removed (signal peptide) | | 26 | |
|  | CamB1 (*Ceanothus americanus)* | frangulanine | | pET28-MBP | | |  |  | Residues M1-G22 removed (signal peptide) | | 26 | |

**Table S2. CamB1 Co-purified Species and Reactivity**

**Corrected concentration of the 50:50 mixture in Gel Fig. S5 (Row 4)*

| **Species** | **Total CamB1 Concentration** | **Percent Conversion** | **Total Turnover (1h)** | **Reference Gel (lane)** |
| --- | --- | --- | --- | --- |
| Cleaved CamB1  **(Minus)**  His_6_-CamB1’ | 1 μM | 19.9% | 29.9 | Fig. S5B (6) |
| His_6_-CamB1’  **(Plus)**  His_6_-CamB1 | 3 μM | 29.8% | 14.9 | Fig. S5A (3) |
| His_6_-CamB1 | 1.5 μM* |  | 29.8 | Fig. S5 |

**Table S3. Exact Masses of Substrate and Product Peptides**

| **Peptides** | **Substrate**  **(Observed m/z)** | **Substrate**  **(Expected m/z)** | **Product**  **(Observed m/z)** | **Product**  **(Expected m/z)** |
| --- | --- | --- | --- | --- |
| **ArbA2-FLWGY** | 744.6973 | 744.6981 | 744.0258 | 744.0262 |
| **CamA1-ILLY** | 731.0277 | 731.0296 | 730.3564 | 730.3577 |

**Table S4. In-Gel Digestion LC-MS/MS Confirmed y/b Fragments (Band 1, Fig. S6)**

| Observed  Unique Peptide | Observed  Monoisotopic Mass | Predicted  Monoisotopic Mass | ΔMass Error (ppm) | Matched Ion m/z  (Observed) | Matched Ion ΔMass Error (ppm) | Matched Ion Counts |
| --- | --- | --- | --- | --- | --- | --- |
| DPSSLPPFLSR | 1214.6315  (608.3230) | 1214.6295 | 1.64 | y1:175.1191  y2:262.1517  y3:375.2361  y4:522.3055  y5:619.3585  y6:358.7092*  y7:829.4953  y8:916.5273  y9:1003.5601  y10:550.810*  b2:213.0874  b3:300.1201  b4:387.1525  b5:500.2373 | 1.33  2.90  3.01  4.00  3.74  3.22  2.75  2.43  3.01  3.42  2.20  3.78  3.99  4.43 | 14 |
| LFSFSQGSHEAK | 1336.6452  (669.3299) | 1336.6411 | 3.09 | y1:147.11228  y2:218.1494  y3:347.1921  y4:484.2511  y5:571.2823  y6:628.3035  y7:756.3628  y8:422.2015*  y9:990.4612  y10:1077.4937  y11:1224.5611  b2:261.1593  b3:348.1917  b5:582.2910  b10:1120.5062 b11:1191.5370 | -3.60  -2.37  -1.05  -0.58  -1.97  -2.12  -0.90  0.38  -2.74  -2.04  -2.62  -1.71  -0.17  -1.95  0.40  -4.9 | 16 |
| TMTVQFFTK | 1101.5546  (551.7846) | 1101.5528 | 1.64 | y1:147.1129  y2:248.1613  y3:395.2309  y4:542.2989  y5:670.3576  y6:769.425  y7:870.4727  y8:501.2610*  b2:233.0961  b3:334.1443 | 1.22  3.55  5.11  3.07  2.69  2.06  0.86  2.26  2.95  3.75 | 10 |
| VGFFTGDDLHVGK | 1390.6929  (464.5715) | 1390.6881 | 3.47 | y1:147.1121  y2:204.1335  y3:303.2018  y4:440.2614  y5:553.3468  y6:668.3703  y7:783.3973  y8:840.4198  y9:471.2383*  y10:544.7710*  b2:157.0963  b3:304.1652  b6:609.3009 | -4.64  -3.45  -2.66  -0.40  2.16  -3.42  -2.83  -1.3  0.75  -2.03  -5.2  -0.9  -3.54 | 13 |

**Table S5. In-Gel Digestion LC-MS/M Confirmed y/b Fragments (Band 2, Fig. S6)**

| Observed  Unique Peptide | Observed  Monoisotopic Mass | Predicted  Monoisotopic Mass | ΔMass Error (ppm) | Matched Ion m/z  (Observed) | Matched Ion ΔMass Error (ppm) | Matched Ion Counts |
| --- | --- | --- | --- | --- | --- | --- |
| DPSSLPPFLSR | 1214.63241 | 1214.6295 | 2.36 | y3:375.2337 y4:522.3034  y5:619.3569  y6:716.4077 y7:829.4908  y8:916.5244  y9:1003.5531  b2:213.0858 b3:300.1182  b4:387.1502 b5:500.2338 | -3.35  -0.02  1.18  -1.79  -2.71  -0.65  -3.91  -5.56  -2.48  -2.08  -2.50 | 11 |
| ESLVEFVSSVLGGR | 1477.78149 (739.8980 m/z) | 1477.7776 | 2.59 | y3:289.1628  y4:402.2446  y6:588.3484  y7:675.3779  y8:774.4478  y9:921.5147  y10:1050.5575  y11:1149.6232  b2:217.0814  b3:330.1658 | 3.24  -3.29  3.44  -0.64  1.37  -0.49  -0.30  -2.57  -1.98  -0.22 | 10 |
| LFSFSQGSHEAK | 1336.64374 (669.3291m/z) | 1336.6411 | 1.93 | y1:147.1123 y2:218.1494 y3:347.1931  y4:484.2509 y5:571.2828 y6:628.3037 y7:756.3625 y8:422.2010* y9:495.7358* y10:1077.4918 y11:612.7851*  b2:261.1598 b3:348.1923  b5:582.2952 b9:991.4585 b10:1120.5013 b11:1191.5371 | -2.82  -2.05  1.81  -1.01  -1.05  -1.82  -1.21  -0.87  0.57  -3.87  -1.08  0.54  1.55  5.12  -4.68  -3.96  -4.86 | 17 |
| PSSTFQNYTFLDVK | 1645.8045 (823.9095 m/z) | 1645.7987 | 3.5 | y1:147.1124  y2:246.1810 y3:361.2087  y4:474.2939  y6:722.4036  y7:885.4686 y10:1274.6479 | -2.21  -0.58  1.58  3.53  -6.44  -3.39  5.05 | 7 |
| TMTVQFFTK | 1101.55392 (551.7842 m/z) | 1101.5528 | 0.95 | y1:147.1126  y2:248.1606 y3:395.2293 y4:542.2976  y5:670.3560 y6:769.4233 y7:870.4700  y8:1001.5102  b2:233.0955  b3:334.1435 | -1.08  0.85  1.07  0.55  0.30  -1.25  -2.24  -2.20  0.51  1.32 | 10 |
| VGFFTGDDLHVGK | 1390.69068 (696.3526 m/z) | 1390.6881 | 1.84 | y1:147.1128  y2:204.1340 y3:303.2026 y4:440.2622 y5:553.3467  y6:668.3716  y7:783.3985  y8:840.4196 y9:941.4650 y10:544.7721*  y11:618.3059* y12:646.8174*  b2:157.0969 b3:304.1659 b4:451.2348  b5:552.2805 b7:724.3295 b11:1188.5650 | 0.13  -0.98  -0.13  1.44  1.98  -1.36  -1.25  -1.63  -3.85  -0.13  -0.78  0.52  -1.63  1.31  1.91  -2.00  -0.71  -2.83 | 18 |

*** Fragmentation ion with 2+ charge**

**References**

(1) Abramson, J.; Adler, J.; Dunger, J.; Evans, R.; Green, T.; Pritzel, A.; Ronneberger, O.; Willmore, L.; Ballard, A. J.; Bambrick, J.; Bodenstein, S. W.; Evans, D. A.; Hung, C. C.; O’Neill, M.; Reiman, D.; Tunyasuvunakool, K.; Wu, Z.; Žemgulytė, A.; Arvaniti, E.; Beattie, C.; Bertolli, O.; Bridgland, A.; Cherepanov, A.; Congreve, M.; Cowen-Rivers, A. I.; Cowie, A.; Figurnov, M.; Fuchs, F. B.; Gladman, H.; Jain, R.; Khan, Y. A.; Low, C. M. R.; Perlin, K.; Potapenko, A.; Savy, P.; Singh, S.; Stecula, A.; Thillaisundaram, A.; Tong, C.; Yakneen, S.; Zhong, E. D.; Zielinski, M.; Žídek, A.; Bapst, V.; Kohli, P.; Jaderberg, M.; Hassabis, D.; Jumper, J. M. Accurate Structure Prediction of Biomolecular Interactions with AlphaFold 3. *Nature* **2024**, *630* (8016), 493-500.

(2) Almagro Armenteros, J. J.; Tsirigos, K. D.; Sønderby, C. K.; Petersen, T. N.; Winther, O.; Brunak, S.; von Heijne, G.; Nielsen, H. SignalP 5.0 Improves Signal Peptide Predictions Using Deep Neural Networks. *Nat. Biotechnol.* **2019**, *37* (4), 420-423.

(3) Teufel, F.; Almagro Armenteros, J. J.; Rosenberg Johansen, A.; Halldór Gíslason, M.; Irby Pihl, S.; Tsirigos, K. D.; Winther, O.; Brunak, S.; von Heijne, G.; Nielsen, H. SignalP 6.0 Predicts All Five Types of Signal Peptides Using Protein Language Models. *Nat. Biotechnol.* **2022**, *40*, 1023-1025.

(4) Lima, S. T.; Pasquale, M. A.; Noyon, M. R. O. K.; Clark, E. A.; Laws, C. R.; Hematian, S.; Chekan, J. R. Peptide Recognition Sequence Guides Catalytic Side Chain Cross-Linking of Plant Peptides by Copper-Dependent Cyclases. *J. Am. Chem. Soc.* **2025**, *147* (24), 20284-20293.

(5) Lima, S. T.; Ampolini, B. G.; Underwood, E. B.; Graf, T. N.; Earp, C. E.; Khedi, I. C.; Pasquale, M. A.; Chekan, J. R. A Widely Distributed Biosynthetic Cassette Is Responsible for Diverse Plant Side Chain Cross‐Linked Cyclopeptides**. *Angew. Chemie Int. Ed.* **2023**, *62* (7), e202218082.

(6) Nielsen, H. Practical Applications of Language Models in Protein Sorting Prediction: SignalP 6.0, DeepLoc 2.1, and DeepLocPro 1.0. In *Methods in Molecular Biology*; Humana Press Inc., 2025; Vol. 2941, pp 153-175.

(7) Shevchenko, A.; Tomas, H.; Havliš, J.; Olsen, J. V; Mann, M. In-Gel Digestion for Mass Spectrometric Characterization of Proteins and Proteomes. *Nat. Protoc.* **2007**, *1* (6), 2856-2860.

(8) MacLean, B.; Tomazela, D. M.; Shulman, N.; Chambers, M.; Finney, G. L.; Frewen, B.; Kern, R.; Tabb, D. L.; Liebler, D. C.; MacCoss, M. J. Skyline: An Open Source Document Editor for Creating and Analyzing Targeted Proteomics Experiments. *Bioinformatics* **2010**, *26* (7), 966-968.

(9) Solntsev, S. K.; Shortreed, M. R.; Frey, B. L.; Smith, L. M. Enhanced Global Post-translational Modification Discovery with MetaMorpheus. *J. Proteome. Res.* **2018**, *17*, 1844-1851.
